# Endothelial jagged-2 sustains hematopoietic stem and progenitor reconstitution after myelosuppression

**DOI:** 10.1172/JCI92309

**Published:** 2017-10-23

**Authors:** Peipei Guo, Michael G. Poulos, Brisa Palikuqi, Chaitanya R. Badwe, Raphael Lis, Balvir Kunar, Bi-Sen Ding, Sina Y. Rabbany, Koji Shido, Jason M. Butler, Shahin Rafii

**Affiliations:** 1 Department of Medicine, Division of Regenerative Medicine, Ansary Stem Cell Institute, Weill Cornell Medicine, New York, New York, USA.; 2 Department of Physiology, Biophysics, and Systems Biology, Weill Cornell Medicine, New York, New York, USA.; 3 Bioengineering Program, DeMatteis School of Engineering and Applied Science, Hofstra University, Long Island, New York, USA.

**Keywords:** Hematology, Vascular Biology, Adult stem cells, Bone marrow, endothelial cells

## Abstract

Angiocrine factors, such as Notch ligands, supplied by the specialized endothelial cells (ECs) within the bone marrow and splenic vascular niche play an essential role in modulating the physiology of adult hematopoietic stem and progenitor cells (HSPCs). However, the relative contribution of various Notch ligands, specifically jagged-2, to the homeostasis of HSPCs is unknown. Here, we show that under steady state, jagged-2 is differentially expressed in tissue-specific vascular beds, but its expression is induced in hematopoietic vascular niches after myelosuppressive injury. We used mice with EC-specific deletion of the gene encoding jagged-2 (*Jag2*) to demonstrate that while EC-derived jagged-2 was dispensable for maintaining the capacity of HSPCs to repopulate under steady-state conditions, by activating Notch2 it did contribute to the recovery of HSPCs in response to myelosuppressive conditions. Engraftment and/or expansion of HSPCs was dependent on the expression of endothelial-derived jagged-2 following myeloablation. Additionally, jagged-2 expressed in bone marrow ECs regulated HSPC cell cycle and quiescence during regeneration. Endothelial-deployed jagged-2 triggered Notch2/Hey1, while tempering Notch2/Hes1 signaling in HSPCs. Collectively, these data demonstrate that EC-derived jagged-2 activates Notch2 signaling in HSPCs to promote hematopoietic recovery and has potential as a therapeutic target to accelerate balanced hematopoietic reconstitution after myelosuppression.

## Introduction

Defining the cellular and molecular components of the bone marrow (BM) niche that regulate the quiescence, self-renewal, and lineage differentiation of hematopoietic stem and progenitor cells (HSPCs) can facilitate targeted interventions toward improved hematopoietic recovery after injury and the expansion of sufficient numbers of HSPCs for transplantation (1, 2). At the core of this dynamic cell-cell interaction is the instructive vascular niche provided by bone marrow endothelial cells (BMECs), which deploy angiocrine signals such as jagged-1 (1–3), Kit ligand (KitL) (1, 2, 4, 5), and CXCL12 (3, 6), to regulate hematopoietic stem cell (HSC) self-renewal and lineage differentiation under both homeostatic and regenerative conditions.

In mammals, endothelial cells (ECs) express 4 types of Notch ligands, jagged-1 (*Jag1*), jagged-2 (*Jag2*), Delta-like 1 (*Dll1*), and Delta-like 4 (*Dll4*) (7). In mice, endothelial-specific knockout of *Jag1* (3) results in the reduction of the number and repopulating capacity of HSPCs at steady state, as well as diminished hematopoietic recovery following myelosuppressive stress. In these mice, the residual Notch activity within HSPCs suggested complementary mechanisms of Notch activation, likely contributed by other Notch ligands, including jagged-2, DLL1, and DLL4. DLL4 haploinsufficiency results in defects in arterial and yolk sac vascular development (8–12). DLL1 was shown to regulate fetal artery development (13). This suggested that DLL1 or DLL4 regulates vascular development in part in a cell-autonomous manner. DLL4 has also been shown to regulate adult hematopoiesis (14). Nonetheless, accumulating evidence demonstrates that signaling afforded by expression of various Notch ligands might perform collectively to induce Notch activation in a dose-dependent manner (15, 16). In this paradigm, the dose of each ligand consummates to induce the level of physiological Notch signaling that ultimately dictates HSPC function. Thus, we hypothesized that the stoichiometry of other Notch ligands, specifically jagged-2 supplied by ECs, might participate in HSPC maintenance by modulating the degree of Notch signaling and HSPC recovery.

To this end, we first characterized the expression of *Jag2* mRNA among adult vascular ECs from different tissue types. In the BM, *Jag2* mRNA and jagged-2 protein are enriched in BMECs compared with non-BMECs. During hematopoietic regeneration, the expression of jagged-2 in BMECs is increased compared with that in homeostatic conditions. Next, using a transgenic mouse line that expresses a *Cre* recombinase under a Cdh5 promoter (17), we deleted exons 1–2 of the *Jag2* gene specifically in ECs (18). This deletion generated a truncated *Jag2* mRNA and truncated jagged-2 protein in ECs. Under steady state, there were minor changes in the hematopoietic indexes in the peripheral blood and in the BM. However, in a more defined EC-HSPC coculture model that mimics HSPC regeneration following myeloablative injury, jagged-2 expressed in ECs was required to promote the HSPC in vitro expansion. Following in vivo myelosuppressive injuries, endothelial jagged-2 preserves the survival rate of mice via maintenance of the HSPCs at both early and later stages of HSPC regeneration. Previous publications suggested that jagged-2 was expressed in both hematopoietic progenitor cells and ECs (19, 20); using transplantation studies, we demonstrated that the engraftment and/or expansion of HSPCs requires endothelial jagged-2. Mechanistically, endothelial jagged-2 induced Notch2/Hey1 signaling and repressed Notch2/Hes1 signaling in HSPCs. The differential requirement of jagged-2 for HSPC function under homeostatic compared with myelosuppressive conditions correlated with the level of jagged-2 expression under these conditions. Indeed, when *Jag2* was deleted from both ECs and hematopoietic cells, there was a more profound alteration of repopulating capacity of HSPCs under steady state conditions. Therefore, jagged-2 serves as an activating module in Notch signaling to promote hematopoietic recovery.

## Results

### Heterogeneity of Jag2 mRNA expression in organotypic ECs.

**Figure 1:**
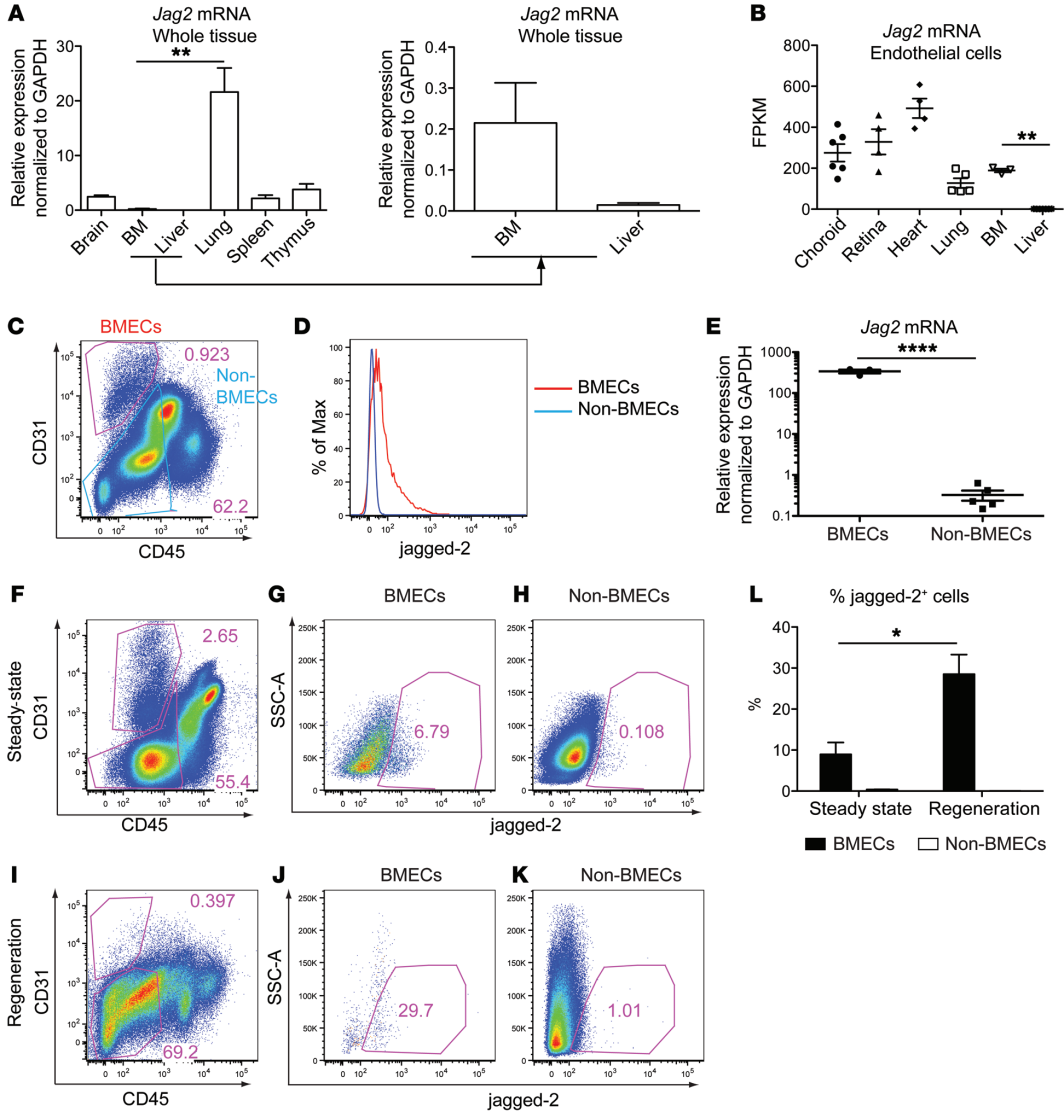
Jagged-2 is dynamically expressed in BMECs. (**A**) The expression level of *Jag2* mRNA in different mouse whole organs (*n* = 3). The mRNA expression is calculated using GAPDH as internal control. (**B**) The FPKM (fragments per kilobase of exon per million fragments mapped) value for *Jag2* mRNA in primary ECs from various organs. The number of dots indicates the number of biological replicates. (**C**) Representative flow cytometric plots for the gating of CD31^+^CD45^–^ BMECs and CD31^–^CD45^–^ non-BMECs (*n* = 4). (**D**) Histogram of jagged-2 expression on BMECs and non-BMECs. (**E**) qPCR quantification of *Jag2* mRNA from sorted BMECs (*n* = 3) and non-BMECs (*n* = 5). The RNA expression level is calculated using GAPDH as internal control. (**F**–**H**) Representative flow plots for jagged-2 expression in BMECs and non-BMECs (*n* = 4) under homeostatic conditions. (**I**–**K**) Jagged-2 expression within BMECs and non-BMECs at 2 weeks after 650 cGy sublethal irradiation (*n* = 5). (**L**) Comparison of percentage of jagged-2^+^ cells among BMECs under steady state and during regeneration after myeloablative injuries. Error bars indicate the SEM. *P < 0.05, **P < 0.01, and ****P < 0.0001, by 2-tailed unpaired *t* test. The numbers in the flow plots represent percentages of cells.

To systematically examine *Jag2* mRNA expression, we carried out reverse transcriptase quantitative PCR (RT–qPCR) in whole tissue lysate from various organs (Figure 1A). *Jag2* mRNA is abundantly expressed in lung, modestly expressed in spleen, thymus, and brain, and expressed at lower levels in BM and liver. Using a previously established protocol (21), we isolated CD45^–^CD31^+^VE-cadherin^+^ primary vascular ECs from various mouse organs and subjected them to RNA sequencing analysis. The expression of *Jag2* mRNA (Figure 1B) was comparable between freshly isolated ECs from lung and BM, suggesting the relative enrichment of *Jag2* expression in BMECs compared with other cell types in the BM. Examination of jagged-2 protein expression pattern in BM via flow cytometry revealed higher expression of jagged-2 in CD31^+^CD45^–^ BMECs than in CD31^–^CD45^–^ non-BMECs (Figure 1, C, D, and F–H). The level of *Jag2* mRNA in sorted BMECs was significantly higher than in non-BMECs (Figure 1E).

When comparing the Notch ligand profile of primary microvascular ECs with cultured ECs, *Jag2* mRNA expression (Supplemental Figure 1A; supplemental material available online with this article; https://doi.org/10.1172/JCI92309DS1) was significantly reduced in the cultured ECs compared with primary isolated choroid ECs, retinal ECs, and lung ECs, but not in BMECs, suggesting that the in vivo microenvironment selectively sustains the expression level of *Jag2* in ECs outside the BM. In contrast, the expression level of *Jag1* mRNA showed either no significant change or increased expression in cultured ECs compared with primary isolated ECs (Supplemental Figure 1B), except for retinal ECs. The expression of *Dll4*, *Dll1*, *Notch1*, and *Notch4* (Supplemental Figure 1, C–F) was also changed in cultured ECs compared with freshly isolated ECs, suggesting an overall change of Notch signaling profiles in cultured versus primary isolated ECs. However, as compared with primary isolated BMECs, the cultured BMECs retained the molecular signatures of *Jag1*, *Jag2*, and *Dll4* expression, with reduction of *Notch1* expression.

In vivo, the level of *Jag2* mRNA expression in ECs also dynamically changed from steady state to myeloablative conditions (Supplemental Figure 1, G–L). During the recovery phase after myelosuppression, the level of *Jag2* and *Dll1* mRNA expression was significantly increased in liver ECs in comparison with steady state (Supplemental Figure 1, G and J). The level of *Dll4* mRNA was significantly decreased, whereas the level of *Jag1*, *Notch4*, and *Notch1* expression did not change, at steady state and following myelosuppression (Supplemental Figure 1, H–L). These data correlate with the previous published microarray data demonstrating the dynamic changes of Notch ligand *Jag2* expression in ECs under steady state and following myeloablative stress (21). In the BM, following myeloablative injuries, the percentage of BMECs expressing jagged-2 was increased at 2 weeks after sublethal irradiation compared with that in homeostatic conditions (Figure 1, I–L), suggesting differential requirement of jagged-2 under steady state and hematopoietic regeneration.

In summary, both *Jag2* mRNA and jagged-2 protein are enriched in BMECs compared with non-BMECs. The dynamic changes of jagged-2 level in BMECs after myelosuppression prompted us to examine the physiological role of endothelial jagged-2 in regulating HSPC homeostasis and regeneration. To this end, we used a conditional knockout mouse model in which the exon 1–2 region of the *Jag2* gene is selectively deleted from ECs, and examined the HSPC behavior under homeostasis and during regeneration.

### Normal angiogenesis in mice with endothelial deletion of Jag2.

To conditionally delete *Jag2* from ECs, we crossed transgenic mice carrying VE-cadherin promoter–driven Cre recombinase (17) with mice carrying *Jag2^fl^* allele (18) that flanked the first 2 exons of *Jag2* gene (Supplemental Figure 2A). We denote the mice with EC-specific deletion of *Jag2* as *Jag2^ECKO^* mice. We next used the reporter analysis (Supplemental Figure 2, B–D) and qPCR analysis of primary isolated ECs (Supplemental Figure 2, E–J) to confirm the deletion efficiency of the VE-cadherin-*Cre* transgenic line (17) in ECs from various vascular beds. The percentage of td-tomato^+^ cells among BMECs was about 89.5%, indicating efficient deletion of VE-cadherin-*Cre*. Via qPCR analysis, we observed that in both BMECs and lung microvascular ECs, the expression level of *Jag2* at the indicated exon regions was reduced by more than 90%; the expression level of the other 3 Notch ligands *Jag1*, *Dll1*, and *Dll4* was not significantly changed (Supplemental Figure 2, H and I). In BMECs, the Notch targets *Hes1* and *Hey1* were reduced when *Jag2* was deleted from BMECs (Supplemental Figure 2J). In lung ECs, the Notch targets *Hes1* and *Hey1* were not significantly changed.

From the RNA sequencing results of primary lung microvascular ECs, we observed that truncated *Jag2* mRNA was generated from *Jag2^ECKO^* mice. The truncated *Jag2* mRNA retained the expression from exons 6–26 (Supplemental Figure 2K). Using 2 antibodies that recognize the N- or C-terminus of jagged-2 protein, respectively, we confirmed that truncated jagged-2 protein was generated in ECs from *Jag2^ECKO^* mice, which lacks the conserved Notch receptor binding DSL and Notch N-terminal (MNNL) domains (ref. 22 and Supplemental Figure 2, L–Q). Most important, we noted that under steady-state conditions there were no apparent defects in the angiogenic profile and vascular perfusion of the *Jag2^ECKO^* mice in the key hematopoietic organs (Supplemental Figure 3). These data indicate that in contrast to *Dll4* deficiency, in which there is a significant defect in angiogenesis, lack of *Jag2* is dispensable for the maintenance of adult organotypic ECs.

### Endothelial jagged-2 is not required for HSPC homeostasis.

**Figure 2:**
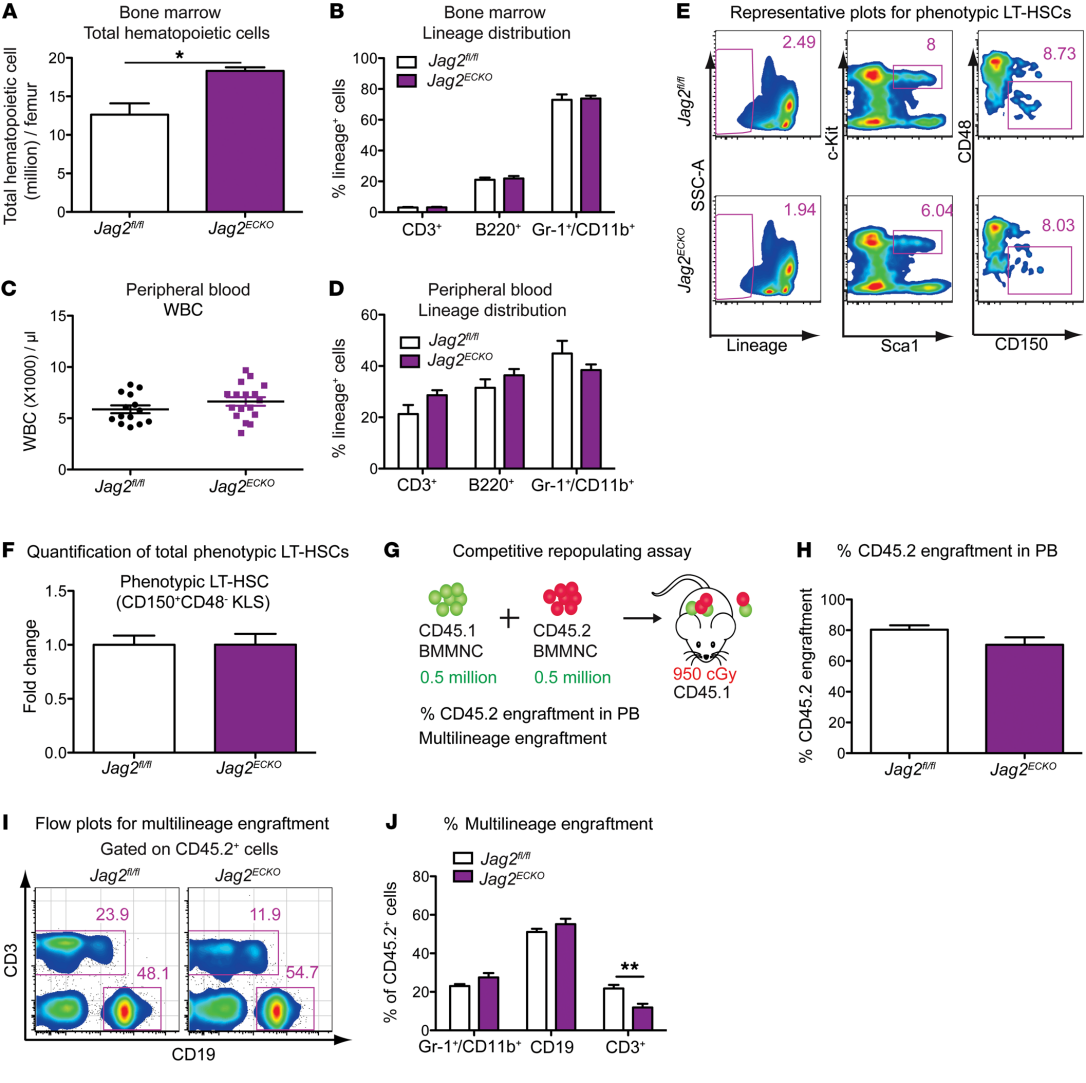
Endothelial-supplied jagged-2 is not required to maintain HSPC function under homeostatic conditions. (**A**–**D**) The total number of BMMNCs (**A**) and the WBC count (**C**) were quantified in adult *Jag2^fl/fl^* or *Jag2^ECKO^* mice. Lineage distribution of hematopoietic cells within the BM (**B**) and peripheral blood (**D**) was quantified in *Jag2^fl/fl^* or *Jag2^ECKO^* mice. For **A** and **B**, *n* = 3. For **C**, the number of dots indicates the number of biological replicates. For **D**, *n* = 9 for *Jag2^fl/fl^*, *n* = 8 for *Jag2^ECKO^*. (**E**) Representative flow cytometric gating of phenotypic Lin^–^c-Kit^+^Sca1^+^CD150^+^CD48^–^ long-term HSCs (LT-HSCs). (**F**) Quantification of the number of phenotypic LT-HSCs per million BMMNCs in the femur of *Jag2^fl/fl^* or *Jag2^ECKO^* (*n* = 8 for each group). (**G**) Schematic view of competitive repopulating assay. (**H**) The percentage of CD45.2^+^ hematopoietic cells in the peripheral blood of CD45.1^+^ mice at 15.5 weeks after transplantation (*n* = 6 CD45.1 recipients for *Jag2^fl/fl^* and *n* = 8 CD45.1 recipients for *Jag2^ECKO^* mice). (**I**) Representative flow cytometric plots showing the multilineage engraftment. The CD45.2^+^ cells were further gated to reveal donor-derived CD3^+^ T cells and CD19^+^ B cells. (**J**) Quantification of multilineage engraftment within the CD45.2^+^ cells. Error bars indicate the SEM. *P < 0.05 and **P < 0.01, by unpaired 2-tailed *t* test. The numbers in the flow plots represent percentages of cells.

We next examined HSPC function under steady-state conditions by quantifying the lineage distributions of hematopoietic cells and the number of phenotypic HSPCs in the BM, followed by competitive transplantation studies. Lack of jagged-2 in the ECs minimally altered the total number of hematopoietic cells in the BM (Figure 2A). The total number of BM mononucleated cells (BMMNCs) was increased in the *Jag2^ECKO^* group (Figure 2A) as compared with *Jag2^fl/fl^* mice. In the peripheral blood, there were no differences in the WBC number in *Jag2^ECKO^* mice compared with *Jag2^fl/fl^* mice (Figure 2C). Closer inspection of the different lineage contributions did not reveal any significant differences in Gr-1^+^/CD11b^+^ myeloid cells, CD3^+^ T cells, or B220^+^ B cells in the BM (Figure 2B) or in the peripheral blood (Figure 2D).

Next, we examined the number and function of the more primitive populations of long-term hematopoietic stem cells (LT-HSCs) defined as Lin^–^c-Kit^+^Sca1^+^CD150^+^CD48^–^ cells (KLS-SLAM) (Figure 2E). There was no difference in the total number of phenotypic LT-HSCs within *Jag2^ECKO^* mice compared with *Jag2^fl/fl^* mice (Figure 2F), suggesting that jagged-2 expressed on ECs was not required to maintain the cell number of primitive LT-HSCs under steady-state conditions.

The repopulating capacity of HSPCs was determined by competitive repopulating assay (Figure 2G). At 15.5 weeks after transplantation (Figure 2H), there were no apparent differences in the percentage of CD45.2^+^ cells in the peripheral blood of CD45.1 mice, demonstrating that BMMNCs from *Jag2^ECKO^* mice contributed to peripheral blood engraftment at a similar level to that of control mice. Multilineage engraftment analysis of the CD45.2^+^ cells revealed a significant reduction in the percentage of T cells contributed from *Jag2^ECKO^* BM cells (Figure 2, I and J) compared with that of the control mice, suggesting the role of endothelial jagged-2 in regulating lymphoid-bias potential of HSPCs (23).

Collectively, the quantification of phenotypic LT-HSCs and functional validation of HSPCs from *Jag2^ECKO^* mice have demonstrated a minimal requirement of EC–jagged-2 in maintaining HSPC number and repopulating capacity under steady state. The lack of obvious HSPC phenotype under steady state suggested that other Notch ligands on ECs or jagged-2 expressed by stromal cells, hematopoietic cells, or other cytokines expressed by the ECs (Supplemental Figure 4) compensate for the loss of function of *Jag2* on ECs. To further reveal the role of endothelial jagged-2 in HSPC function, a more defined in vitro coculture system using BMECs and HSPCs was carried out to test the hypothesis that EC–jagged-2 is necessary for the in vitro expansion of HSPCs. Subsequently, in vivo myelosuppressive models were used to reveal the role of endothelial jagged-2 in promoting HSPC regeneration following myelosuppressive stress.

### Jagged-2 expressed on BMECs is required for in vitro expansion of HSPCs.

**Figure 3:**
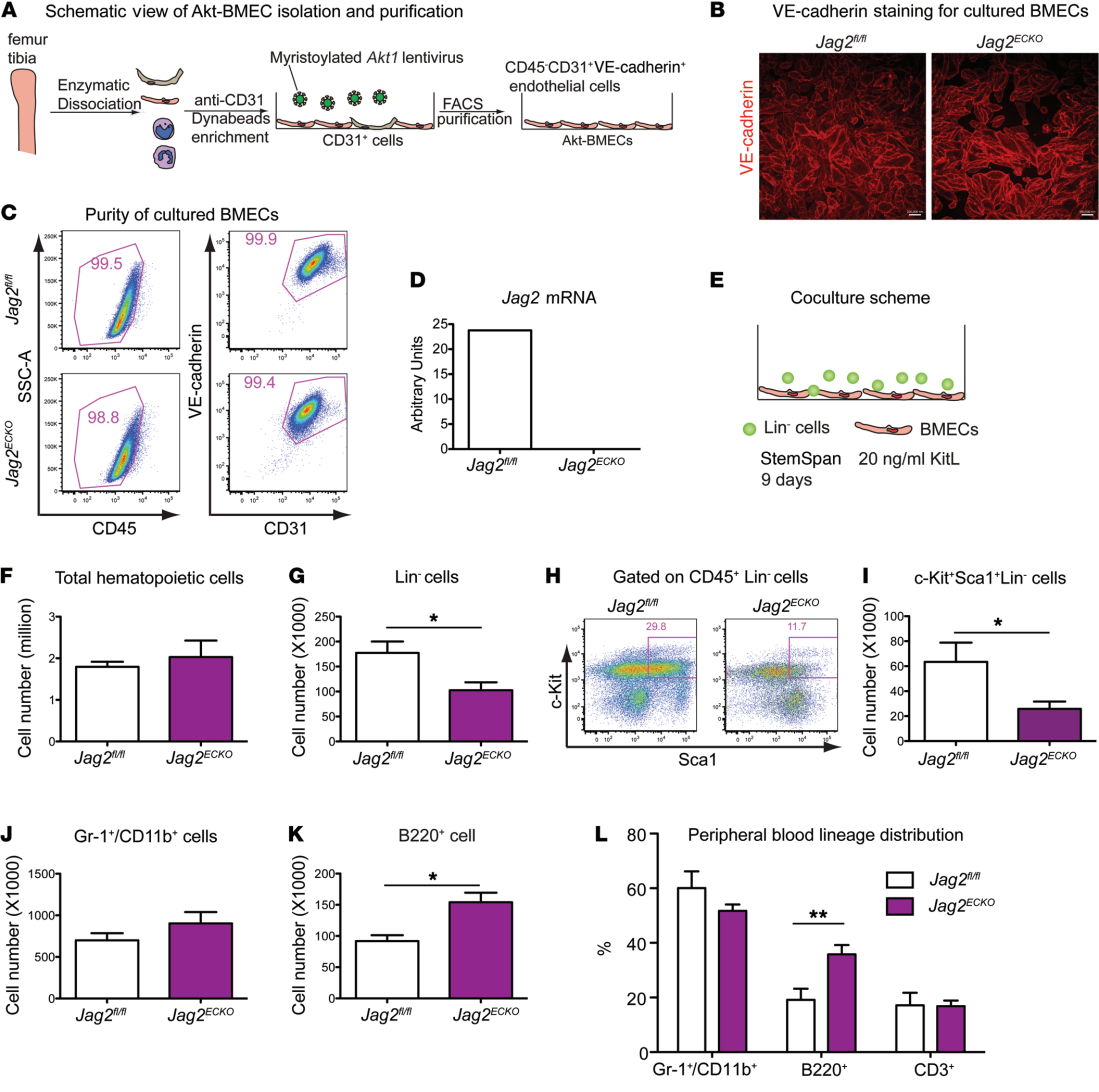
Jagged-2 expressed by BMECs promotes in vitro expansion of HSPCs. (**A**) Schematic view of the method to enrich BMECs from *Jag2^fl/fl^* or *Jag2^ECKO^* mice. (**B**) The cultured BMECs were stained with anti–VE-cadherin antibody. Scale bars: 100 μm. (**C**) After passages, the purity of BMECs from *Jag2^fl/fl^* or *Jag2^ECKO^* mice was confirmed by flow cytometric staining of CD45, CD31, and VE-cadherin (*n* = 5). The percentage of CD45^–^CD31^+^VE-cadherin^+^ ECs is shown. (**D**) Using primers flanking the first 2 exons of *Jag2* mRNA, the expression level of *Jag2* was quantified via real-time qPCR (*n* = 3). (**E**) Schematic view of the coculture setup using BMECs and lineage-negative (Lin^–^) hematopoietic cells (*n* = 3 biological replicates of Lin^–^ cells were used for the coculture; 1 *Jag2^fl/fl^* BMEC and 2 lines of *Jag2^ECKO^* BMECs were used as feeders). (**F**–**I**) At day 9 after coculture, the total number of CD45^+^ cells (**F**), Lin^–^ cells (**G**), and KLS cells (**I**) within the culture was summarized. The representative flow cytometric plots of KLS HSPCs are shown in **H**. (**J** and **K**) At day 9 after coculture, the total number of Gr-1^+^/CD11b^+^ myeloid cells (**J**) and B220^+^ cells (**K**) was quantified. (**L**) Quantification of peripheral blood multilineage distribution in 18-month-old *Jag2^fl/fl^* or *Jag2^ECKO^* mice (*n* = 7 for *Jag2^fl/fl^*, *n* = 8 for *Jag2^ECKO^*). Error bars indicate the SEM. *P < 0.05 and **P < 0.01, by 2-tailed *t* test. The numbers in the flow plots represent percentages of cells.

A more defined in vitro coculture assay that modeled hematopoietic regeneration was used to dissect the role of jagged-2 on BMECs in promoting the expansion of HSPCs (Supplemental Figure 5A). To this end, we enriched BMECs from *Jag2^fl/fl^* mice or *Jag2^ECKO^* mice (3, 17). Dynabead-based cell enrichment (24) was carried out using an mAb directed to mouse CD31 (Figure 3A). After cell separation, lentivirus-encoding myristoylated *Akt1* gene was transduced into the primary cells, and we term these cells Akt-BMECs. Following a minimum of 5 cell passages and additional CD31^+^VE-Cadherin^+^ cell sorting to ensure purification, Akt-BMECs from both control and *Jag2^ECKO^* mice displayed cobblestone pattern with contact inhibition and robust VE-cadherin expression (Figure 3B). The survival and proliferation of BMECs from *Jag2^ECKO^* mice were not significantly different from those of the control mice (data not shown). After several passages, the identity and purity of BMECs were maintained (Figure 3C); more than 97% of the cultured BMECs were CD45^–^CD31^+^VE-cadherin^+^ ECs. RT–qPCR analysis using primers flanking exons 1–2 of *Jag2* mRNA demonstrated that *Jag2* mRNA was abundantly expressed in *Jag2^fl/fl^*, and its expression was almost undetectable in BMECs from *Jag2^ECKO^* mice (Figure 3D). Western blot confirmed loss of the N-terminus of jagged-2 protein in the in vitro cultured BMECs (Supplemental Figure 2N).

Following the coculture of Lin^–^ cells on BMECs derived from *Jag2^fl/fl^* or *Jag2^ECKO^* mice (Figure 3E), the total numbers of CD45^+^ hematopoietic cells, Lin^–^ cells, and HSPCs were quantified. Though there were no significant differences between the numbers of total expanded CD45^+^ hematopoietic cells (Figure 3F), there was a significant reduction in the number of expanded Lin^–^ cells (Figure 3G) and KLS HSPCs (Figure 3, H and I, and Supplemental Figure 5, B–D) when Lin^–^ cells were cocultured on BMECs from *Jag2^ECKO^* compared with *Jag2^fl/fl^* mice. Loss of jagged-2 on BMECs reduced HSPC expansion to 40% of that expanded on control BMECs. To determine whether jagged-2 on BMECs modulates HSPC differentiation, the total of expanded cells was examined by antibody staining with Gr-1 and CD11b for myeloid cells and B220 for B cells. The number of expanded Gr-1^+^/CD11b^+^ myeloid cells was not significantly different when Lin^–^ cells were cocultured on BMECs from *Jag2^fl/fl^* or *Jag2^ECKO^* mice (Figure 3J). The total number of B220^+^ cells was significantly increased when Lin^–^ cells were cultured on BMECs derived from *Jag2^ECKO^* mice, compared with BMECs from *Jag2^fl/fl^* mice (Figure 3K and Supplemental Figure 5E). Thus, jagged-2 on BMECs prevented the differentiation of Lin^–^ cells or HSPCs into B220^+^ cells. Indeed, in a long-term analysis to reveal the role of endothelial jagged-2 in regulating HSPC differentiation, we observed that within the peripheral blood of 18-month-old *Jag2^ECKO^* mice, the percentage of B220^+^ B cells was significantly increased compared with that in *Jag2^fl/fl^* mice (Figure 3L and Supplemental Figure 5F), suggesting increased differentiation of HSPCs into B cells (25). Taken together, these results suggest that jagged-2 supplied by BMECs maintains HSPCs by inhibiting the differentiation of Lin^–^ cells or HSPCs into B220^+^ cells.

### Endothelial jagged-2 promotes recovery of HSPCs after myelosuppression.

**Figure 4:**
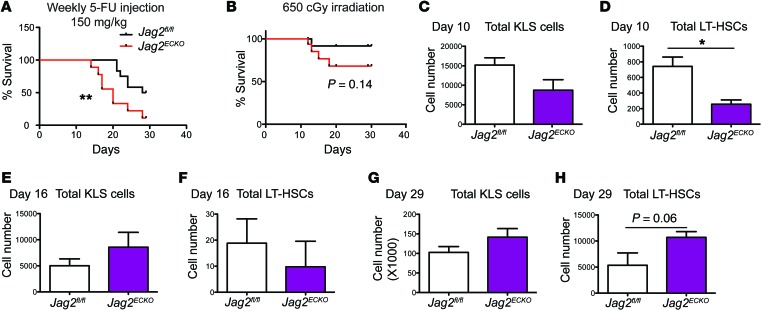
Endothelial jagged-2 ensures proper HSPC recovery after myelosuppression. (**A**) *Jag2^fl/fl^* or *Jag2^ECKO^* mice were subjected to weekly 5-fluorouracil (5-FU) injections at the dose of 150 mg/kg. Kaplan-Meier survival curve was generated after monitoring of the survival rate of the mice. *n* = 12 for *Jag2^fl/fl^*, *n* = 9 for *Jag2^ECKO^* mice. (**B**) *Jag2^fl/fl^* or *Jag2^ECKO^* mice were subjected to sublethal irradiation at 650 cGy, and their survival rate was monitored. This experiment was performed 4 times. Each time, *n* = 4 mice were used for each genotype. The data are combined and shown in **B**. (**C**–**H**) To monitor the kinetics of hematopoiesis regeneration following irradiation, the quantification of KLS cells and LT-HSCs at day 10 (**C** and **D**) (*n* = 4 for each genotype), day 16 (**E** and **F**) (*n* = 5 for each group), and day 29 (**G** and **H**) (*n* = 5 for each group) after sublethal irradiation is further shown. Error bars indicate the SEM. *P < 0.05 and **P < 0.01, by unpaired 2-tailed *t* test.

The coculture model demonstrates the requirement of jagged-2 to expand HSPCs in vitro. To test the role of endothelial jagged-2 in protecting hematopoietic reconstitution after myelosuppression, 2 regimens were used to cause myeloablative injuries to mice. One involves weekly injection of the chemotherapy reagent 5-fluorouracil; the other is γ-irradiation administered at a sublethal dose of 650 cGy. Eight- to ten-week-old *Jag2^fl/fl^* mice or *Jag2^ECKO^* mice were subjected to weekly 5-flurouracil injections. The survival rate of the mice was monitored weekly (Figure 4A). The whole cohort of *Jag2^ECKO^* mice died faster and earlier than the cohort of control mice. There was a reduced survival rate of *Jag2^ECKO^* mice compared with *Jag2^fl/fl^* mice (Figure 4A). This result underscores the protective role of endothelial jagged-2 in preventing myelosuppressive damage.

We further tested whether endothelial jagged-2 could protect the mice from sublethal irradiation (26). Following 650 cGy sublethal irradiation, WT mice undergo a regeneration phase that lasts about 4–6 weeks (24). In a combination of 4 batches of mice (a total of 16 mice were used for each genotype), more *Jag2^ECKO^* mice died than control mice (Figure 4B). There was a trend toward reduction of survival rate of *Jag2^ECKO^* mice compared with that of the control mice. Because the death of mice was detected around day 12 after irradiation in the *Jag2^ECKO^* mice, and on the basis of the regeneration kinetics observed in previous publications (3, 26), we examined the hematopoietic recovery in the BM on day 10, day 16, and day 29 after irradiation. During the recovery of the hematopoietic reconstitution, there was a slight decrease in the number of RBC and platelets in the *Jag2^ECKO^* mice compared with *Jag2^fl/fl^* control mice, yet the WBC count did not show significant differences between the 2 groups (Supplemental Figure 6, A–E). At day 10, the total number of hematopoietic cells within the BM of *Jag2^fl/fl^* or *Jag2^ECKO^* was similar; however, the more primitive KLS cells (Figure 4C) showed a trend toward reduction in *Jag2^ECKO^* mice compared with *Jag2^fl/fl^* mice. The analysis of LT-HSCs revealed a significant decrease in the number of LT-HSCs at day 10 after irradiation in *Jag2^ECKO^* mice compared with *Jag2^fl/fl^* mice (Figure 4D).

At day 16 after irradiation, the total numbers of hematopoietic cells, Lin^–^ cells, KLS cells, and LT-HSCs were the lowest among the 3 time points examined, correlating with the lowest number of WBC, RBC, and platelets in the peripheral blood (Supplemental Figure 6, A–C) and the need for rapid regeneration of KLS cells at this stage of recovery. At day 16 after irradiation, there were no significant differences between the KLS numbers (Figure 4E and Supplemental Figure 6, F and G) or between the LT-HSC numbers (Figure 4F) in *Jag2^fl/fl^* and *Jag2^ECKO^* mice. At day 29, the number of LT-HSCs showed a trend toward increase in *Jag2^ECKO^* mice compared with *Jag2^fl/fl^* mice (Figure 4, G and H).

**Figure 5:**
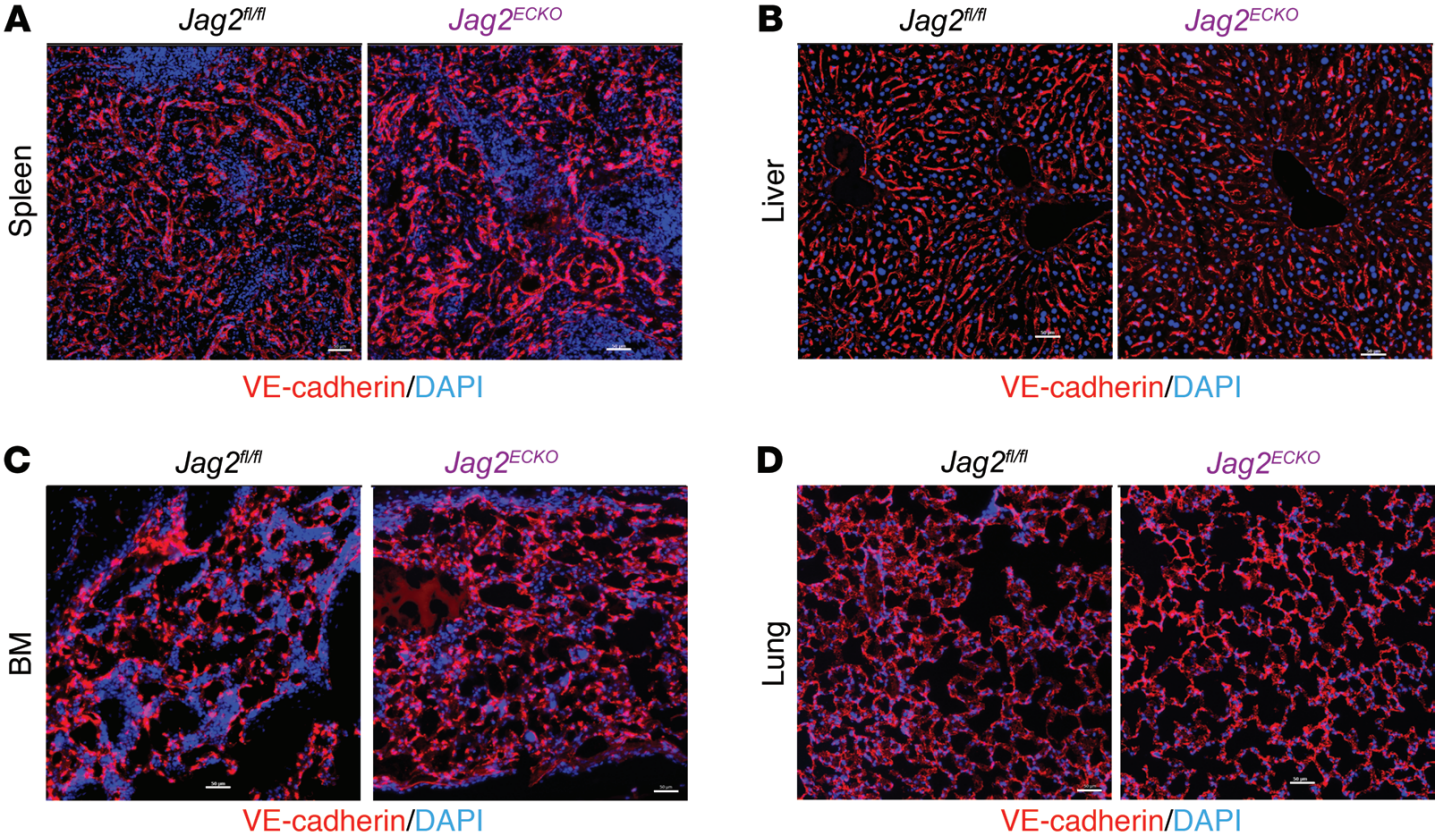
Vascular perfusion function is preserved in *Jag2^ECKO^* mice following myelosuppression. At day 10 after 650 cGy irradiation, the perfusion function of spleen vasculature (**A**), liver vasculature (**B**), BM vasculature (**C**), and lung vasculature (**D**) was preserved in *Jag2^ECKO^* mice, as demonstrated by fluorescence-labeled VE-cadherin antibodies perfused into the vasculature (*n* = 4 for each group). Scale bars: 50 μm.

As Notch signaling regulates vascular sprouting angiogenesis and arterial maintenance (16), we examined whether the reduced number of phenotypic LT-HSCs in *Jag2^ECKO^* mice was due to defective vasculature functions. At day 10 after 650 cGy irradiation, hematopoietic organs including spleen (Figure 5A), liver (Figure 5B), and BM (Figure 5C) were examined for vascular perfusion function. Vasculature was well perfused in the spleen and liver in *Jag2^ECKO^* mice compared with *Jag2^fl/fl^* mice, suggesting that there were no vascular patterning defects or perfusion abnormalities in those organs. In the BM, VE-cadherin staining indicated preservation of perfusion functions of BM of *Jag2^ECKO^* mice and *Jag2^fl/fl^* mice. In nonhematopoietic organs such as lung (Figure 5D), there were slight alterations of lung alveolar structures, with staining of VE-cadherin in *Jag2^ECKO^* mice compared with control mice.

**Figure 6:**
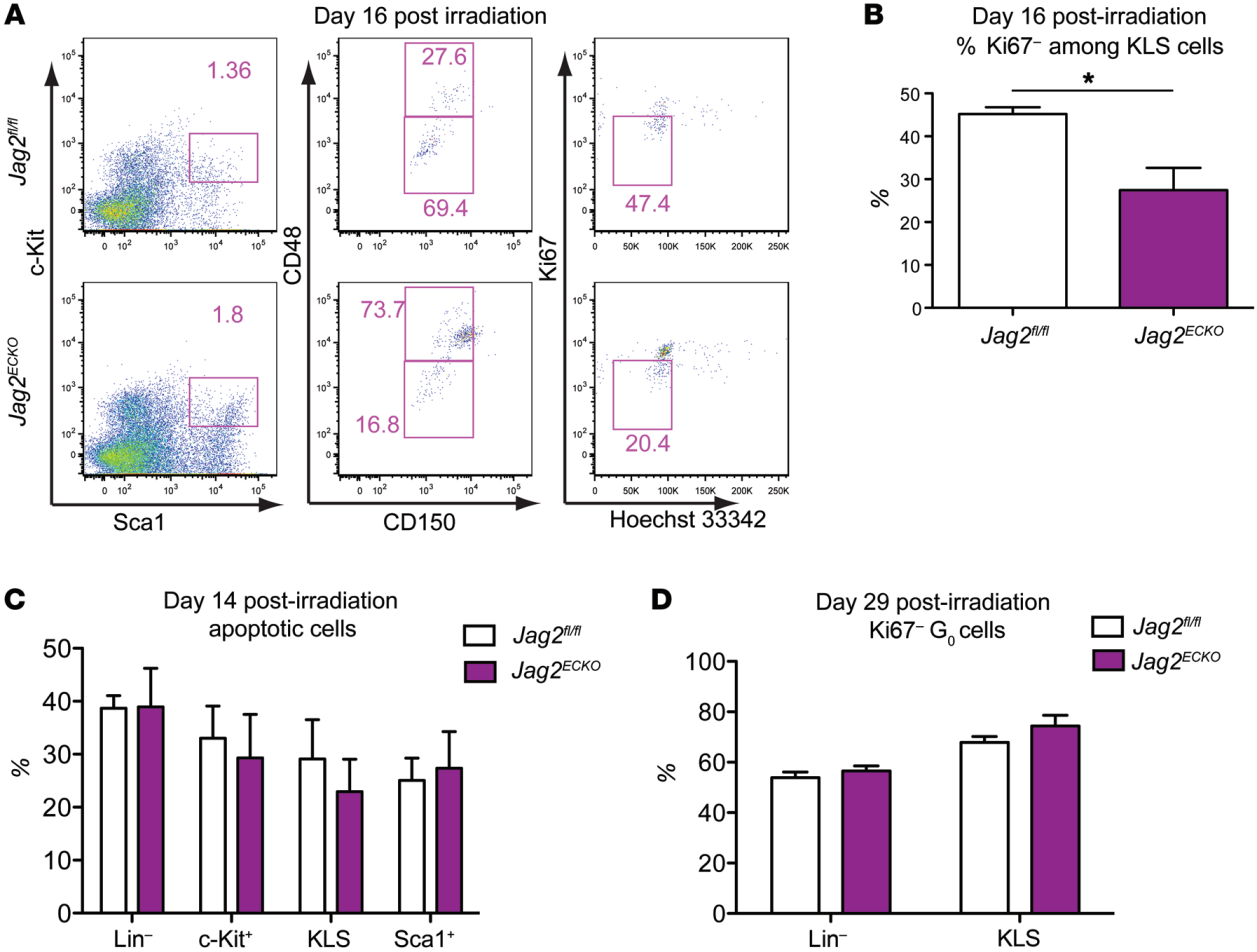
Endothelial jagged-2 modulates the cell-cycle status of HSPCs during regeneration. (**A**) On day 16 after irradiation, the flow cytometric gating for KLS, CD150^–^CD48^–^ short-term KLS (ST-HSCs), and CD150^–^CD48^+^ KLS multipotent progenitor cells is shown. (**B**) At day 16 after irradiation, the percentage of Ki67^–^ G_0_ cells among KLS cells was quantified (*n* = 5 for each group). (**C**) At day 14 after irradiation, the percentage of apoptotic cells among Lin^–^ cells, cKit^+^Lin^–^ cells, KLS cells, and Sca1^+^Lin^–^ cells was quantified. (**D**) On day 29 after 650 cGy irradiation, the percentage of Ki67^–^ G_0_ cells among Lin^–^ cells and KLS cells was quantified (*n* = 5 for each group). Error bars indicate the SEM. *P < 0.05, by unpaired 2-tailed *t* test. The numbers in the flow plots represent percentages of cells.

We next examined the cell cycle status of the KLS cells at day 16 and day 29 after irradiation. On day 16, when the least number of KLS cells and LT-HSCs resided in the BM, only 45.2% of KLS cells remained at the Ki67^–^G_0_ quiescent stage in *Jag2^fl/fl^* mice (Figure 6, A and B), compared with 70% at G_0_ stage at day 29 after 650 cGy irradiation (Figure 6D). At day 16, there was significant reduction of percentage (27.42%) of Ki67^–^ G_0_ stage quiescent KLS cells in *Jag2^ECKO^* mice compared with *Jag2^fl/fl^* (45.2%) mice (Figure 6, A and B), suggesting the requirement of endothelial jagged-2 to maintain the fine balance between quiescence and proliferation of KLS cells. The apoptosis rate of KLS cells at day 14 after irradiation was similar between the 2 groups (Figure 6C and Supplemental Figure 6, H and I). At day 21, there was a significant increase in both the forward scatter and the side scatter (Supplemental Figure 6, J and K) of KLS cells from *Jag2^ECKO^* mice compared with *Jag2^fl/fl^* mice, suggesting the altered morphology of KLS cells during regeneration when jagged-2 is deleted from ECs. At day 29, the difference between the cell cycle status of KLS cells was diminished between *Jag2^ECKO^* and *Jag2^fl/fl^* mice (Figure 6D).

Taken together, the kinetics of total hematopoietic cells, Lin^–^ cells, KLS cells, and LT-HSCs after sublethal irradiation indicated that although the number of total hematopoietic cells did not reduce to almost undetectable at day 16 after irradiation, the more primitive cells such as KLS cells and LT-HSCs had undergone a rapid exhaustion (day 0 to day 16). This was followed by a decision of KLS cells to delicately balance between their quiescent stem cell identity and rapid proliferation to replenish the progenitor cell pool (day 10 to day 16), and they finally returned to a state similar to steady-state conditions (~day 29 and onward). Therefore, the decrease of LT-HSC number in the BM of *Jag2^ECKO^* mice at day 10 indicates that loss of jagged-2 correlates with reduced HSPC maintenance at the early stage of myelosuppression. At the mid-phase that precedes the rapid regeneration, loss of endothelial jagged-2 causes rapid proliferation of KLS cells, which leads to the upward trend in LT-HSCs at the final stage of hematopoietic regeneration.

Previous studies of the microvascular EC gene expression profiles before and after 650 cGy sublethal irradiation have revealed significant upregulation of *Jag2* mRNA in BMECs at day 10 after irradiation compared with steady state levels. However, the level of *Jag2* expression gradually fell to steady-state level, as indicated by reduced *Jag2* mRNA expression in liver ECs and BMECs at day 28 after irradiation compared with day 10 after irradiation (21). These data supported the critical role of endothelial jagged-2 in mediating hematopoietic recovery after irradiation, and the notion that the biological outcome of the loss of function of *Jag2* correlated with its dose of expression in specific biological contexts.

### Engraftment of HSPCs is not dependent on the Notch2/Hes1 signaling axis in HSPCs.

**Figure 7:**
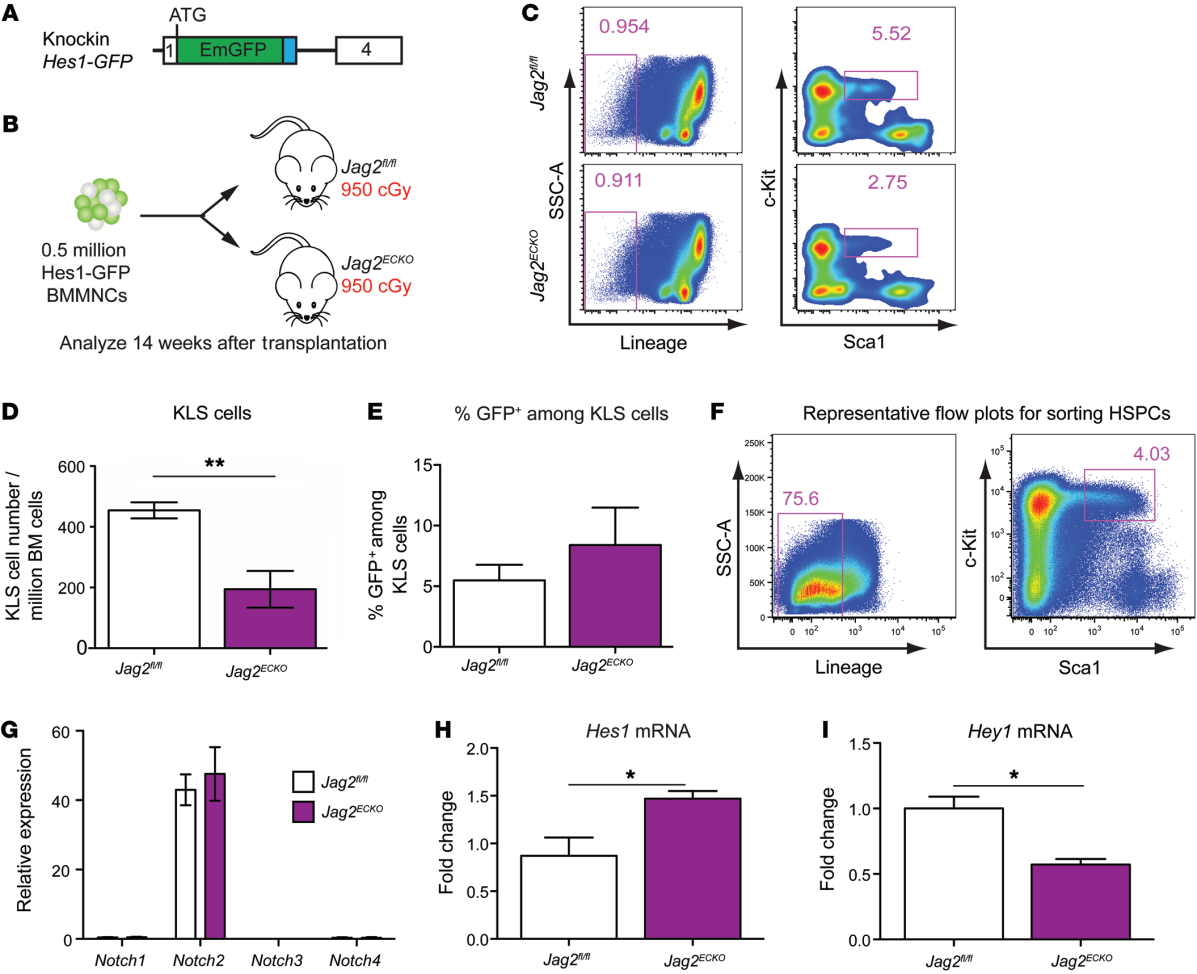
Endothelial jagged-2 induces *Notch2/Hey1* signaling in HSPCs, enhancing engraftment. (**A** and **B**) Schematic view of the knockin *Hes1-GFP* reporter mouse line (**A**) and the transplantation assays (**B**) used to test the role of BMEC jagged-2 in promoting engraftment/expansion of HSPCs. *n* = 4 for *Jag2^fl/fl^* group, *n* = 3 for *Jag2^ECKO^* group. (**C**) Representative flow plots for the gating strategies for KLS cells in the *Jag2^fl/fl^* and *Jag2^ECKO^* mice. (**D**) The number of HSPCs per million BM cells was quantified. (**E**) The percentage of GFP^+^ cells among the gated KLS cells is summarized. The data were collected following the transplantation experiment listed in **B**. (**F**) Representative flow cytometric plots showing the sorting strategies for KLS cells from *Jag2^fl/fl^* or *Jag2^ECKO^* mice. (**G**–**I**) Real-time qPCR analysis of the sorted HSPCs from *Jag2^fl/fl^* or *Jag2^ECKO^* mice was carried out for Notch receptors (**G**) and Notch targets (**H** and **I**). For **G** and **I**, *n* = 3 biological replicates for each group. For **H**, *n* = 5 for each group. Error bars indicate the SEM. *P < 0.05 and **P < 0.01, by unpaired 2-tailed *t* test. The numbers in the flow plots represent percentages of cells.

Jagged-2 is expressed on both ECs and hematopoietic cells (19). To test whether endothelial jagged-2 is necessary for engraftment and/or expansion of HSPCs, we used an experimental model in which an equal number of BMMNCs was transplanted into lethally irradiated *Jag2^fl/fl^* or *Jag2^ECKO^* mice (Figure 7B). Specifically, we took advantage of *Hes1-GFP* knockin reporter (27, 28) mice to visualize the activation status of *Hes1* via flow cytometry (Figure 7A). Fourteen weeks after the transplantation of *Hes1-GFP* BM mononuclear cells into *Jag2^fl/fl^* or *Jag2^ECKO^* mice, the number of HSPCs per million total hematopoietic cells in the BM of *Jag2^ECKO^* mice was significantly reduced compared with that in control mice (Figure 7, C and D), suggesting the requirement of endothelial jagged-2 in maintaining HSPC number after transplantation. Loss of jagged-2 on ECs caused reduction of c-Kit^+^Sca1^+^Lin^–^ HSPCs by 58% in comparison with *Jag2^fl/fl^* mice. There was no significant difference in the percentage of Hes1^+^ cells within KLS cells that were transplanted into *Jag2^ECKO^* mice compared with *Jag2^flfl^* mice (Figure 7E).

We next aimed to delineate the Notch signaling pathways involved in angiocrine communication between vascular ECs and HSPCs (3) by identifying the Notch receptors and Notch downstream targets expressed in HSPCs in both *Jag2^flfl^* and *Jag2^ECKO^* mice. To this end, we sorted out KLS HSPCs (Figure 7F) from *Jag2^fl/fl^* mice and *Jag2^ECKO^* mice under steady state. Via RT–qPCR analysis, we confirmed that the Notch receptors expressed in HSPCs from control mice were mainly *Notch2*, with undetectable expression of *Notch1*, *Notch3*, or *Notch4* (Figure 7G). These data correlated with the recent publication using Notch receptor promoter–driven *Cre* to map out the Notch receptor expression in the hematopoietic system (27). There were no changes of Notch receptor expression levels in *Jag2^ECKO^* mice compared with the control mice. For the Notch downstream targets, there was a significant reduction of the Notch target *Hey1* in sorted HSPCs in *Jag2^ECKO^* mice compared with *Jag2^fl/fl^* mice (Figure 7I), suggesting that endothelial jagged-2 activated the Notch2/Hey1 axis in HSPCs. Notably, loss of jagged-2 in ECs caused upregulation of *Hes1* level in the HSPCs both under steady state (Figure 7H) and during hematopoietic regeneration following irradiation (Supplemental Figure 6L), suggesting the role of endothelial jagged-2 in curbing the expression of *Hes1* in HSPCs.

Collectively, these data demonstrated the non–cell-autonomous role of endothelial jagged-2 in maintaining HSPC cell number following myelosuppressive regimens. For the downstream signaling events, endothelial jagged-2 activates the Notch2/Hey1 signaling axis in HSPCs, and balances the upward trend of Notch2/Hes1 signals in HSPCs. The unique dichotomy of *Hey1* and *Hes1* signals in HSPCs might lead to as-yet unrecognized specific changes in HSPC behaviors, which will manifest under various physiological or stress situations.

### Endothelial and hematopoietic cell–derived jagged-2 maintains HSPC number.

**Figure 8:**
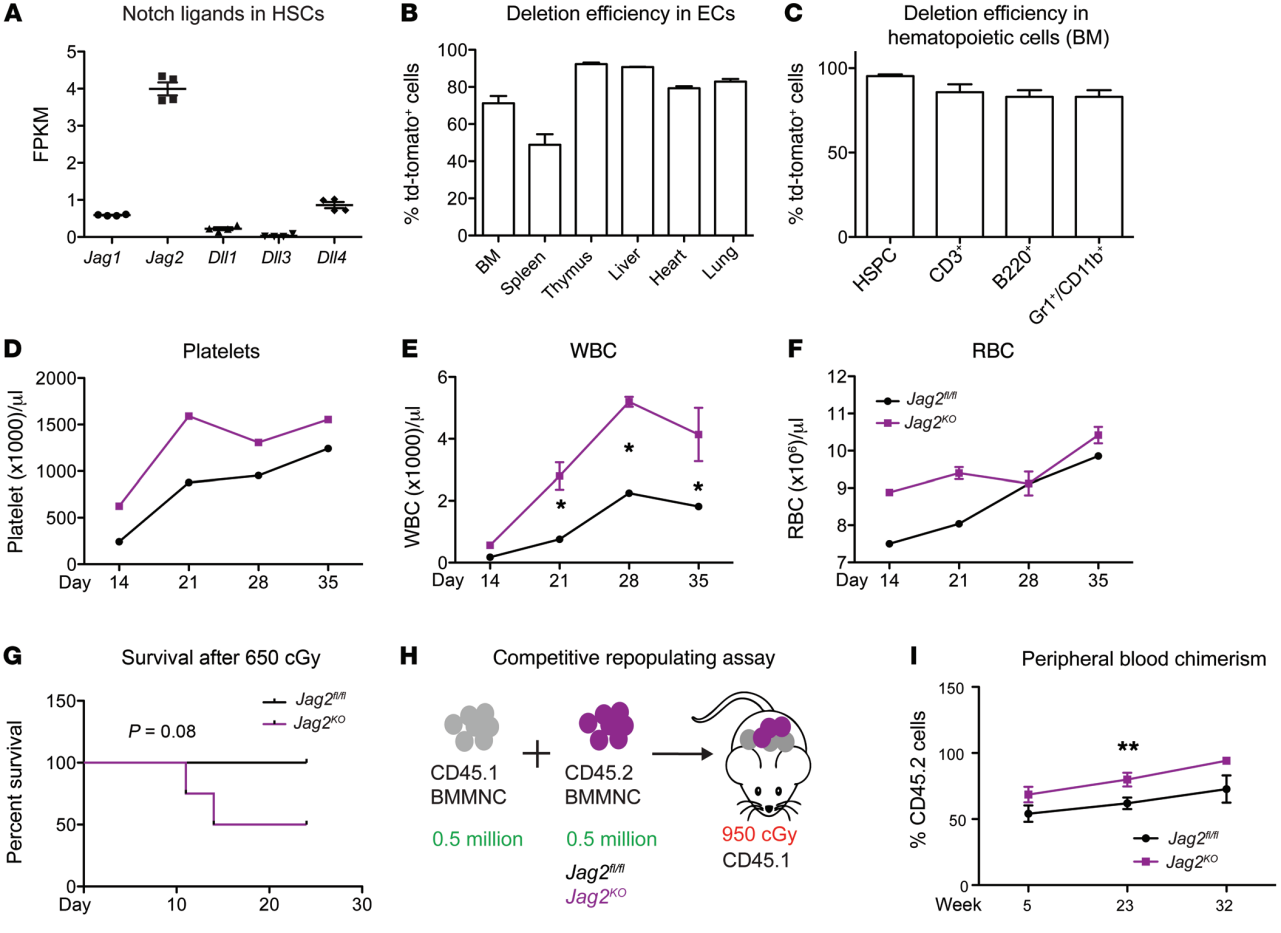
Jagged-2 supplied by ECs and hematopoietic cells maintains HSC number. (**A**) Summary of mRNA expression of Notch ligands *Jag1*, *Jag2*, *Dll1*, *Dll3*, and *Dll4* in HSPCs. Data are from the published RNA sequencing data of SP-KLS-CD150^+^ cells (29). VE-cadherin-*Cre* mice (30) were crossed with *Jag2^fl/fl^* mice to delete exons 1 and 2 of the *Jag2* gene from ECs and hematopoietic cells. (**B** and **C**) After crossing of VE-cadherin-*Cre* mice with *Rosa26^CAG<stop>tdtomato^* mice, the deletion efficiency of VE-cadherin-*Cre* in ECs (**B**) and hematopoietic cells within the BM (**C**) was quantified. (**D**–**F**) The platelets (**D**), WBC (**E**), and RBC (**F**) were monitored after sublethal irradiation with 650 cGy at the indicated time points. (**G**) The survival curve of *Jag2^fl/fl^* or *Jag2^KO^* mice was plotted. (**H** and **I**) Competitive repopulating assay was carried out using BMMNCs from *Jag2^fl/fl^* or *Jag2^KO^* mice (*n* = 7 for each group). For **B** and **C**, *n* = 3 biological replicates for each group. For **D**–**G**, *n* = 5 for *Jag2^fl/fl^*, *n* = 4 for *Jag2^KO^* mice. For **I**, *n* = 7 each for *Jag2^fl/fl^* and *Jag2^KO^* mice. Error bars indicate SEM. For **D**–**F**, at each individual time point, the difference between *Jag2^fl/fl^* and *Jag2^KO^* mice was compared using a 2-tailed *t* test. The resulting *P* value is shown. For **D**–**F** and **J**, the overall differences of the 2 curves were also compared using 2-way ANOVA, and the *P* values of the observed variance based on the genotype are as follows: for **D**, *P* = 0.0025; for **E**, *P* < 0.0001; for **F**, *P* = 0.0373; for **I**, *P* = 0.0016. *P < 0.05; **P < 0.01 (**P value was determined by 2-way ANOVA).

It was previously reported that jagged-2 is also expressed in HSPCs (refs. 19, 29, and Figure 8A). To investigate the consequence of compound deletion of *Jag2* from both ECs and hematopoietic cells, we used another VE-cadherin-*Cre* transgenic line (30), which was shown to recombine in ECs of various organs and hematopoietic cells (Figure 8, B and C). We denote the resulting VE-cadherin-*Cre*^+/–^ *Jag2^fl/fl^* mice as *Jag2^KO^* mice, to distinguish them from the *Jag2^ECKO^* mice (Supplemental Figure 2) used for the majority of this study.

The deletion of *Jag2* from ECs and hematopoietic cells led to increased WBC count following myelosuppressive injury (Figure 8, D–F) with a trend of reduced survival for the *Jag2^KO^* mice compared with *Jag2^fl/fl^* mice (Figure 8G). When an equal number of BMMNCs from *Jag2^KO^* mice or *Jag2^fl/fl^* mice were transplanted into lethally irradiated CD45.1 mice, BMMNCs from *Jag2^KO^* mice resulted in elevated peripheral blood chimerism into CD45.1 host mice (Figure 8, H and I), suggesting the role of jagged-2 in regulating HSPC repopulating capacity. This suggested a dose effect of jagged-2 as Notch ligand; when *Jag2* was deleted from both hematopoietic cells and ECs (Figure 8G), the resulting interference with marrow reconstitution was more profound than when *Jag2* was deleted only from ECs (Figure 4B).

## Discussion

Balanced self-renewal and fine-tuned differentiation of HSPCs are essential for the proper reconstitution of hematopoiesis. While numerous niche-derived pathways have been shown to choreograph HSPC regeneration, the precise mechanism(s) by which niche signals drive long-term regeneration of the HSPCs is being actively scrutinized and refined. Proper activation of Wnt, BMP, and Notch signaling pathways conveyed by the niche cells, such as the vascular niche, orchestrates the hematopoietic reconstitution. Among these pathways, the Notch signaling pathway plays a seminal role in mandating HSPC self-renewal at the expense of differentiation, thereby safeguarding long-term hematopoiesis. However, the mechanism by which Notch pathways mastermind hematopoietic recovery is complex and depends on coordinated and tissue-specific angiocrine expression of Notch ligands such as jagged-2. Here, we have shown that jagged-2 represents an essential arm of Notch signaling that ensures proper HSPC recovery after myeloablative injury.

### Contribution of Notch signaling to hematopoietic recovery.

Notch signaling was shown to mediate the regeneration of long-term repopulating HSPCs following myelosuppressive conditions such as 5-fluorouracil treatment (31). Notch signaling enhanced the expansion of murine HSPCs by ECs (32, 33). Therefore, the Notch signaling was required for HSPC regeneration and in vitro expansion.

The differential contribution of Notch ligands expressed by ECs to HSPCs during in vitro expansion and regeneration is not well studied. Mouse ECs express 4 types of Notch ligands, jagged-1, jagged-2, DLL1, and DLL4. It was previously shown that immobilized Notch ligand DLL1 in combination with hematopoietic cytokines expands the absolute number of HSPCs, and that those expanded hematopoietic progenitor cells engraft into BM of immunodeficient NOD-SCID mice (34). EC-specific deletion of *Dll4* increased tip cell formation in retinal angiogenesis, suggesting autocrine function of DLL4 in regulating angiogenesis and vascular remodeling (8, 16). Similarly, *Dll1* is expressed in embryonic arteries and is required to induce VEGF-A expression in arteries (13). Postnatally, expression of DLL1 on ECs ensures arteriogenesis in hind limb ischemia models. Therefore, DLL4 and DLL1 might primarily be engaged in neoangiogenesis.

Because of the apparent vascular defects in *Dll4*- or *Dll1-*knockout mice, we used conditional knockout mouse models (*Jag1^ECKO^* or *Jag2^ECKO^*) in which the perfusion and vascular patterning were not severely affected to examine the perfusion-independent role of ECs in HSPC homeostasis and reconstitution. Indeed, in *Jag1^ECKO^* mice (3), vascular patterning was changed minimally compared with that in control mice. However, the number of HSPCs was reduced under steady state and hematopoietic regeneration was delayed following myelosuppressive stresses. Furthermore, residual HSPC function and Notch activity within HSPCs in the *Jag1* conditional knockout mice suggested a complementary mechanism of Notch activation. To this end, we hypothesize that the other member of the mammalian serrate Notch ligands expressed on ECs, jagged-2, complements jagged-1 and maintains HSPCs during homeostasis and reconstitution.

### Jagged-2/Notch regulation of steady-state and stressed hematopoiesis.

Our results indicate that jagged-2 is differentially expressed among vascular beds. In the BM, *Jag2* mRNA and jagged-2 protein are enriched in ECs. Jagged-2 supplied by ECs is not required for HSPC maintenance under steady state. Here, we show that jagged-2 is required for HSPC expansion in BMEC-mediated in vitro coculture experiments. Moreover, jagged-2 expression on ECs maintains HSPC reconstitution following myelosuppressive conditions. Although hematopoietic cells also express jagged-2, the maintenance and engraftment of HSPCs into irradiated BM is dependent on endothelial jagged-2. Finally, jagged-2 triggers Notch2/Hey1 signaling in HSPCs to maintain HSPC function during recovery following myelosuppressive stress.

These lines of evidence indicate that endothelial jagged-2 performs as a functional Notch ligand and induces Notch signaling between ECs and HSPCs. Since under steady state, HSPCs undergo self-renewal and differentiation at a physiological rate; it is plausible that upon loss of jagged-2, other Notch ligands, jagged-1, DLL1, or DLL4, are compensating for Notch activation. It is also conceivable that jagged-2 expressed by BM stromal cells or hematopoietic cells (Figure 8A) or other pro-HSPC angiocrine factors in the BM niche (Supplemental Figure 4) compensates for loss of function of jagged-2 on ECs. In contrast, the in vitro EC-HSPC coculture model and HSPC regeneration following myelosuppressive stresses represent processes where rapid HSPC recovery accompanied by HSPC self-renewal, expansion, and differentiation takes places within a narrow time window, and concerted actions of a plethora of angiocrine factors are required to maintain the fine balance between HSPC self-renewal and HSPC exhaustion. In line with the requirement of Notch signaling in maintaining HSPC reconstitution, the expression of Notch ligands in key hematopoietic organs such as BMECs and liver ECs was increased in the time window corresponding to rapid HSPC recovery following myelosuppressive stress (ref. 21, Figure 1L, and Supplemental Figure 1G). This can also explain the more dramatic HSPC phenotype observed following myelosuppressive stress compared with steady state. Alternatively, it is possible that during regenerative processes, the role of Notch ligands in angiogenesis was more apparent, and the observed lethality phenotype associated with loss of the Notch ligand jagged-1 or jagged-2 on ECs might be due to a vascular defect or perfusion impairment. To identify the key event leading to defective HSPC recovery, we quantified HSPC number during the recovery phase (Figure 4, C–H), and monitored vascular perfusion function via i.v. injection of fluorescence-labeled VE-cadherin antibodies (Figure 5, A–D). Histological sections showed minor changes in vascular structures and preservation of vascular perfusion function as demonstrated by VE-cadherin staining of key organs involved in HSPC recovery. Nonetheless, the number of LT-HSCs was significantly reduced at the early phase of myelosuppressive responses (Figure 4D), and during the mid-recovery phase, the KLS cells failed to go back to quiescence as efficiently when jagged-2 was deleted from ECs (Figure 6B).

The observed HSPC phenotype might be also due to the differential regulation of downstream Notch targets in HSPCs induced by jagged-2 or jagged-1 expression on ECs. Notably, jagged-1 induced both *Hey1* and *Hes1* expression in HSPCs, and jagged-2 induced *Hey1* activation in HSPCs (Figure 7I). The ability to influence multiple Notch downstream targets by EC–jagged-1 (3) would likely result in more drastic perturbation of HSPC function when *Jag1* was deleted from ECs, consistent with the observation that under steady state, HSPC number was reduced in *Jag1^ECKO^* mice but remained unchanged in *Jag2^ECKO^* mice. *Hes1* expression in HSPCs of *Jag2^ECKO^* mice is increased in comparison with control mice. Given the recent findings that increased Notch activity caused rapid proliferation of premature exhaustion of bona fide HSPCs and increased lymphoid differentiation (35), it is conceivable that jagged-2 expression on ECs curbs the expression of *Hes1* on HSPCs to ensure proper balance of downstream Notch targets in HSPCs and maintains HSPC self-renewal and differentiation.

We note that because *Jag2^fl/fl^* mice were maintained on a B6/129 background, the studies using *Jag2^ECKO^* and *Jag2^KO^* mice bear the caveat that, regardless of absence of rejection, strain inconsistencies might induce phenotypes that could be responsible for the differences attributed to the specific deletion discussed above (Figures 2 and 4–8). We have repeated these experiments several times to diminish potential bias introduced by minor strain differences.

In summary, our data set forth the new finding that jagged-2 is a functional Notch ligand in which its angiocrine expression by the vascular niche promotes HSPC reconstitution under myelosuppressive conditions without altering vascular perfusion. Moreover, angiocrine expression of jagged-2 by triggering Notch2/Hey1, augments engraftment and expansion of HSPCs. Future experimental studies are needed to dissect the role of Notch2/Hey1 activation induced by EC–jagged-2 in promoting HSPC maintenance. Collectively, these data lay the foundation for employing jagged-2 expression as a therapeutic modality to accelerate balanced hematopoietic recovery after myelosuppression.

## Methods

### Mice.

VE-cadherin-*Cre* mice were obtained from the laboratories of M.L. Iruela-Arispe (Department of Molecular, Cell, and Developmental Biology, University of California, Los Angeles, CA, USA) and N.A. Speck (Abramson Family Cancer Research Institute and Department of Cell and Developmental Biology, University of Pennsylvania, Philadelphia, PA, USA) and were maintained on a B6 background. *Jag2^fl/fl^* mice were obtained from the laboratory of T. Gridley (Center for Molecular Medicine, Maine Medical Center Research Institute, Scarborough, ME, USA) and were of B6/129 background.

### Cell lines.

Cultured choroid ECs, retinal ECs, lung ECs, and liver ECs were from Angiocrine Biosciences. The ECs were authenticated via flow cytometric staining of CD45^–^CD31^+^VE-cadherin^+^ markers. The cells were tested free of mycoplasma.

### Generation of BMEC lines.

Ten microliters per animal of Dynabeads (Dynabeads Sheep Anti-Rat IgG; Invitrogen; 11035) was prepared 1 day before the EC isolation. The proper amount of Dynabeads was taken out and washed in MACS buffer (PBS + 2 mM EDTA + 0.1% BSA + 1% penicillin/streptomycin/antimitotic). Dynabeads were then incubated with CD31 antibody for more than 1 hour to overnight. Age- and sex-matched adult *Jag2^fl/fl^* or *Jag2^ECKO^* mice were used. Two femurs and 2 tibiae from hind limb were dissected out. The attached muscle was cleared first by scissors and then by Kimwipes (Kimberly-Clark Worldwide, Inc.). The femurs and tibiae were then grounded using sterile mortars and pestles. The crude BM solution was then digested using collagenase/dispase at a working concentration of 2.5 mg/ml (Roche 11088793001 for collagenase A, 04942078001 for Dispase II) for 15 minutes at 37°C on an orbital shaker. Following the enzymatic dissociation, the reaction was stopped by addition of DMEM plus 10% FBS. The cells were then filtered through a 40-μm cell strainer to obtain single-cell suspension. The cells were then incubated with anti-CD31–coated Dynabeads for 45 minutes at 4°C on a rotation shaker. After the incubation, the Dynabeads-bound cells were washed 5 times in MACS buffer. The cells were then grown on a fibronectin-coated 12-well plate using mouse EC complete medium. Three days later or when the cobblestone structure of mouse ECs appeared, lentivirus-encoding myristoylated Akt1 was transduced into ECs, and cells were grown to full confluence, which was achieved within a month. Following passages and expansion, BMECs were further purified using FACS sorting. Frozen stocks of early passages of BMECs were prepared for future use. The staining of cultured BMECs using VE-cadherin antibody (R&D Systems) was carried out to verify the purity and morphology of BMECs. The VE-cadherin staining was repeated at least 6 times to verify the ECs.

### Cell culture.

BMECs were grown in mouse EC complete medium. To prepare 500 ml of mouse EC complete medium, 200 ml of F-12 medium (Corning, Cellgro; 10-080-CV) and 200 ml DMEM low-glucose medium (Corning, Cellgro; 10-014-CV) were mixed with 100 ml heat-inactivated FBS. The medium was then supplemented with 5 ml nonessential amino acid (Corning, Cellgro; 25-025-CI), 5 ml of penicillin/streptomycin/amphotericin (Corning, Cellgro; 30-004-CI), 10 ml of 1 M HEPES (Corning, Cellgro, 25-060-CI), 5 ml of 10 mg/ml heparin stock (Sigma-Aldrich; H3149-100KU), and 5 ml of 7.5 mg/ml EC growth supplement (Alfa Aesar J64516). Cells were passed every 3 days or whenever cells reached confluence. Freezing medium was prepared by mixing of 4:1 volume of heat-inactivated FBS and DMSO (Sigma-Aldrich). Cells were pelleted and resuspended into mouse EC complete medium. An equal volume of freezing medium was added into the cell suspension in a dropwise manner. To thaw cells, a frozen tube was immediately put into a 37°C incubator. After transfer of the cells into a 15-ml Falcon tube, mouse EC complete medium was added dropwise into the cell suspension. The same procedure was repeated to remove residual DMSO. Cells were then plated on tissue culture flasks.

### In vitro BMEC-HSPC coculture assays.

BMECs isolated from *Jag2^fl/fl^* control mice or *Jag2^ECKO^* mice were seeded into a 12-well plate and grown into confluence in mouse EC complete medium. Lin^–^ hematopoietic cells were isolated from BM of adult mice. The same number of BM Lin^–^ cells were added into each well on top of BMECs and cultured in defined medium StemSpan (Stemcell Technologies) supplemented with knockout serum replacement and 20 μg/ml mouse Kit-ligand (stem cell factor) (PeproTech). Every other day 1 ml of fresh medium was added into the coculture well; following the fourth day, the floating hematopoietic cells were collected and spun down and transferred into a new confluent layer of BMECs. Fresh medium was added to the old well of BMECs with attached hematopoietic cells. Following the split event, fresh medium was added to both the new well and old wells every other day. At 9 days after coculture, all the cells in the wells were collected after enzymatic dissociation. The cells were then separated into CD45^hi^ hematopoietic cells and CD45^dim^ hematopoietic or ECs. For each cell population, cells were stained with the hematopoietic marker CD45, lineage antibodies, and the HSPC markers c-Kit and Sca1. Cell counting beads were added before flow cytometric analysis. The total number of hematopoietic cells, Lin^–^ cells, and HSPCs was quantified by normalizing to the number of counting beads and the gating frequencies. The total numbers of hematopoietic cells, Lin^–^ cells, and KLS cells were added from both the CD45^dim^ and CD45^hi^ populations.

### Competitive transplantation assays.

Ten- to twelve-week-old CD45.1 mice were purchased from The Jackson Laboratory. The CD45.1 mice were lethally irradiated at 950 cGy 1 day before transplantation. On the day of transplantation, 1 femur was dissected out from *Jag2^fl/fl^* or *Jag2^ECKO^* mice. Muscles were detached from the femur. After cutting of the 2 ends of the femur, BM was flushed out using a 26G needle infused with sterile PBS. The BM cell suspension was filtered through a 40-μm cell strainer. After cell counting, 0.5 million CD45.2^+^ BMMNCs were combined with 0.5 million CD45.1^+^ BMMNCs and were transplanted into lethally irradiated CD45.1 mice. At the indicated time points after transplantation (Figure 2H and Figure 8I), peripheral blood was examined by flow cytometric staining to check the percentage of CD45.2 engraftment; lineage antibodies were also stained to check the multilineage engraftment and lineage differentiation potential of the engrafted HSPCs.

### Flow cytometric analysis to analyze competitive repopulating assay.

To draw blood, mice were anesthetized using an isoflurane chamber at a constant flow rate of vaporized isoflurane 3-5% for induction and adjust the oxygen to the flow rate of 0.5-1.0L/ml. Two caliber tubes of blood were drawn from retro-orbital veins of mice and added into 200 ml of 10 mM EDTA/PBS buffer. After brief vortexing, the sample was put on ice until all the blood samples were collected. RBC were then lysed using 5 ml of RBC Lysis Buffer (BioLegend; 420301) on ice. The cells were then transferred into an Eppendorf tube for staining. Cells were incubated with mouse FcR block in 50 μl MACS buffer for 10 minutes. Antibodies for Ter119, CD45.1, CD45.2, CD3, B220, and Gr-1/CD11b were then mixed to generate a master mix and added into each tube. The information for the antibodies is listed in Supplemental Table 1. For all the experiments, unstained sample or fluorescence-minus-one (FMO) rule was used for gating of positive populations. Unstained control and single-stained cells or ultracompensation beads (eBioscience; 01-2222-42) were used to calculate the fluorescence spillover from all the channels. Compensation was calculated either manually or automatically and applied to all the samples.

### Flow cytometric analysis to quantify LT-HSC number and cell cycle status.

Age- and sex-matched adult *Jag2^fl/fl^* or *Jag2^ECKO^* mice were used for this quantification. Femurs and tibiae from mice were dissected. Following muscle cleanup, the femurs and tibiae were ground using mortars and pestles in MACS buffer. The cells were then filtered through a 40-μm cell strainer; the remaining bones were ground twice more following the same procedure until the bones appeared white. The total number of filtered cells was counted as the total hematopoietic cells (excluding mature RBC) in the BM. Two million total hematopoietic cells were then stained with lineage antibodies and the SLAM-HSPC markers c-Kit, Sca1, CD150, and CD48. The total number of SLAM-HSPCs was calculated using the total number of hematopoietic cells and the frequencies of Lin^–^ cells and of c-Kit^+^Sca1^+^ and CD150^+^CD48^–^ gating. For steady-state HSPC quantification, this same method was used. For quantification of HSPC recovery kinetics following myelosuppression, total hematopoietic cells were counted. Lineage depletion was then carried out. The Lin^–^ cells were stained with SLAM-HSPC markers. The total number of SLAM-HSPCs was quantified using the Lin^–^ cell number and the frequencies of c-Kit^+^Sca1^+^ and CD150^+^CD48^–^ gating. For cell cycle analysis of the SLAM-HSPCs, following surface staining of c-Kit, Sca1, CD150, and CD48, the cells were washed in MACS buffer and fixed in 1% paraformaldehyde (PFA) for 3 minutes. Following intracellular permeabilization (BD Biosciences; Cytofix/Cytoperm), the cells were stained with Ki67. Finally, the cells were washed of excessive Ki67 staining and incubated with DAPI overnight. WT Lin^–^ cells that had undergone the same surface staining and intracellular permeabilization were used as FMO gating control. Ultracompensation beads were stained with each antibody for compensation purposes.

### Perfusion function of vasculature at steady-state conditions or after 650 cGy irradiation.

Monoclonal antibody that targets mouse VE-cadherin (BioLegend; clone BV13) was conjugated with Alexa Fluor 647 dye (Life Technologies, Thermo Fisher Scientific) in the laboratory. At day 10 after sublethal irradiation (650 cGy), 25 μg conjugated VE-cadherin antibodies was retro-orbitally injected into *Jag2^fl/fl^* control or *Jag2^ECKO^* mice. Ten minutes after antibody injection, the mice were euthanized with CO_2_. Organs such as liver, lung, heart, BM, and spleen were dissected out and washed in PBS. After fixation in 4% PFA, tissues were dehydrated in 30% sucrose, embedded in OCT compound, and snap-frozen. Tissue sections (10-μm-thick) were then prepared using a cryostat machine. The tissue sections were counterstained with DAPI to reveal the nuclei. Pictures were taken using a Zeiss LSM 710 confocal microscope. For the imaging of mouse BM vasculature, more than 3 images were taken for the vasculature near the growth plate; more than 5 images were taken for the metaphysis region. For image analysis, the indicated numbers of fields were randomly chosen. The data from the different image fields were then averaged as the vessel density of the organ of interest. The investigators were unaware of the genotype of mice when recording the images.

### FACS sorting of primary ECs.

Monoclonal antibody that targets mouse VE-cadherin (Biolegend, clone BV13) was conjugated with Alexa Fluor 647 dye (Life Technologies) in the laboratory. Age- and sex-matched *Jag2^fl/fl^* or *Jag2^ECKO^* mice were used for EC isolation and RNA preparation. Twenty-five micrograms of conjugated VE-cadherin antibodies was retro-orbitally injected into *Jag2^fl/fl^* control or *Jag2^ECKO^* mice. Ten minutes after antibody injection, the mice were euthanized with CO_2_. For BMEC isolation, femurs and tibiae were dissected, then ground with mortars and pestles, and then enzymatically digested with collagenase/dispase mixture at 37°C for 15 minutes. Following retrieval of single-cell suspension, lineage depletion was carried out using a lineage cell depletion kit (Miltenyi Biotec). Lineage-negative cells were then stained with the hematopoietic and EC surface markers CD45 and CD31. DAPI was stained lastly in the buffer of PBS plus 2 mM EDTA. BMECs were then sorted as DAPI^–^CD45^–^CD31^+^VE-cadherin^+^ cells.

For isolation of ECs from choroid, retina, lungs, and livers, 25 μg isolectin and 25 μg VE-cadherin were injected into mice. After tissue dissection, the tissues were first cut into small pieces using razor blades in a sterile Petri dish. The small tissue pieces were then enzymatically digested using collagenase/dispase mixture at 37°C for 30 minutes on an orbital shaker. Following enzymatic digestion, the cells were then further broken into homogenous single-cell suspension via titration using a 21-gauge needle attached to a syringe. The enzymatic reaction was then stopped via neutralization by FBS. The RBC within the single-cell suspension were lysed using RBC lysis buffer (BioLegend). The cells were then resuspended in MACS buffer and stained with the EC marker CD31. Microvascular ECs were sorted also as CD45^–^isolectin^+^VE-cadherin^+^ cells.

### Quantitative real-time PCR analysis and sample preparation of RNA sequencing analysis.

For whole-organ *Jag2* mRNA expression analysis, a piece of tissue from lung, liver, or BM was homogenized in RNA lysis buffer, RLT buffer (Qiagen) with 1% β-mercaptoethanol. The RLT solution was then cleared of undigested tissue using a QIAthredder column (Qiagen). RNA was then prepared following the RNeasy protocol (QIAGEN). DNA was digested using DNase I (QIAGEN) during the RNA extraction processes. The obtained RNA was measured for concentration using NanoDrop (Thermo Fisher Scientific). RNA (200 ng) was used for cDNA conversion using qScript Super Mix (Quanta Biosciences). After cDNA dilution with double-distilled H_2_O at a 1:5 ratio, the qPCR reaction was set up by mixing the gene-specific primers and SYBR Green qPCR Reaction Mix (KAPA Biosystems). qPCR was run and detected using a ViiA-7 qPCR machine (Life Technologies, Thermo Fisher Scientific). Please refer to Supplemental Table 2 for the exact primer sequence(s) for indicated gene. For RNA extraction of abundant cells, a QIAGEN RNeasy kit was used. For RNA extraction of low numbers of cells following FACS sorting, the cells were pelleted down and lysed using extraction buffer from PicoPure RNA Isolation Kit (Life Technologies, Thermo Fisher Scientific). DNA was removed during RNA extraction processed with DNase I (Qiagen). For RNA sequencing analysis, the extracted RNA was submitted to the Genomic Core Facility within Weill Cornell Medicine. The RNA concentration and integrity were measured using a NanoDrop and Agilent 2100 Bioanalyzer (Agilent), respectively. The integrity of RNA was indicated by the RNA integrity number (RIN). RNA samples with sufficient concentration and RIN greater than 8.0 were further prepared for cDNA library preparation and subsequent sequencing. Refer to GSE95835 (Gene Expression Omnibus, NCBI) for RNA sequencing results of primary choroid and retinal ECs.

### Western blot.

For Western blotting of tissue of *Jag2^fl/fl^* or *Jag2^ECKO^* mice, small pieces of tissue were homogenized using automatic tissue homogenizer (Qiagen) in RIPA buffer that contained protease inhibitor, phosphate inhibitor, and sodium orthovanadate. Tissues were lysed in this buffer on ice for 1 hour. After sonication, supernatant was collected for protein concentration measurement and Western blot analysis. Protein concentration was measured using a BCA assay kit (Thermo Fisher Scientific). Protein sample was then mixed with loading buffer and reduced using 5% β-mercaptoethanol. The protein sample was boiled at 95°C for 10 minutes before being loaded onto SDS-PAGE gel. Membrane transfer was done using wet transfer technique. Primary antibody was incubated with 5% milk in PBST (PBS+0.2% Tween-20) buffer overnight at the dilution of 1:1,000. Secondary antibodies were incubated at room temperature for 1 hour. Signals were amplified and detected using an ECL kit using x-ray films. For the Western blotting of lung tissues, more than 2 separate batches of experiments were performed (each time, *n* = 2 for *Jag2^fl/fl^* or *Jag2^ECKO^* mice).

### Immunofluorescent staining of cultured cells.

For immunofluorescent staining of cultured cells, cells were removed from growth medium and washed with PBS once. The cells were then fixed in 4% PFA for 10 minutes at room temperature. After blocking with blocking buffer, cells were incubated with primary antibody overnight. Secondary antibodies were stained the next day. Finally, cells were counterstained using Hoechst 33328 nuclear dye. After washes with PBS, cells were then incubated with PBS before images were taken using a Zeiss LSM 710 confocal microscope.

### Statistics.

The differences in the phenotypic analysis of experimental parameters were statistically compared using a 2-tailed *t* test. For measurements that were made across different time points between the 2 genotypes, 2-way ANOVA was carried out to measure the statistical significance of the observed data for the 2 factors, time and genotype; the *P* value of the observed phenotype variance based on genotype was reported and considered for statistical significance. *P* less than 0.05 was considered to be statistically significant; *P < 0.05, **P < 0.01, and ***P < 0.001. Experiments were repeated at least 2–3 times. The variance was similar between the groups being statistically compared. If higher variability was found, we increased the sample size to fully confirm statistical significance. No statistical method was used to predetermine sample size for a given effect size. We included all tested animals for quantification to analyze statistical difference.

### Study approval.

All animal experiments were performed under the approval of Weill Cornell Medicine Institutional Animal Care and Use Committee, New York, NY. The breeding and maintenance of animal colonies abided by the guidelines of the IACUC of Weill Cornell Medical College, New York, New York, USA. All experimental procedures followed the IACUC guidelines. Genotyping was carried out in the laboratory or the tails were sent to Transnetyx (transnetyx.com). To compare the phenotypes between different mouse genotypes, sex- and weight-matched littermates were used. Mice at 20–28 g body weight were used. Investigators who performed the experiments and recorded the data were blinded to genotypes of individual mice.

## Author contributions

PG designed the study, performed the experiments, interpreted the results, and wrote the paper. MGP, BP, CRB, RL, BK, and BSD performed the experiments and analyzed the data. BP, CRB, and BSD performed mouse experiments and collected and analyzed the data. BSD, SYR, KS, and JMB helped to formulate the hypothesis, interpreted the results, and edited the manuscript. PG and SR conceived the project, analyzed the data, and wrote the paper.

## Supplementary Material

Supplemental data

## Version 1 10/23/2017

Electronic publication

## Version 2 12/01/2017

Print issue publication
