# Supplementary material for: Endothelial jagged-2 sustains hematopoietic stem and progenitor reconstitution after myelosuppression
Source: J Clin Invest. 2017 Oct 23;127(12):4242–56. doi: 10.1172/JCI92309 (PMC5707154; doi:10.1172/JCI92309)
Supplement: Supplemental data [file jci-127-92309-s001.pdf]

SUPPLEMENTARY FIGURE 1

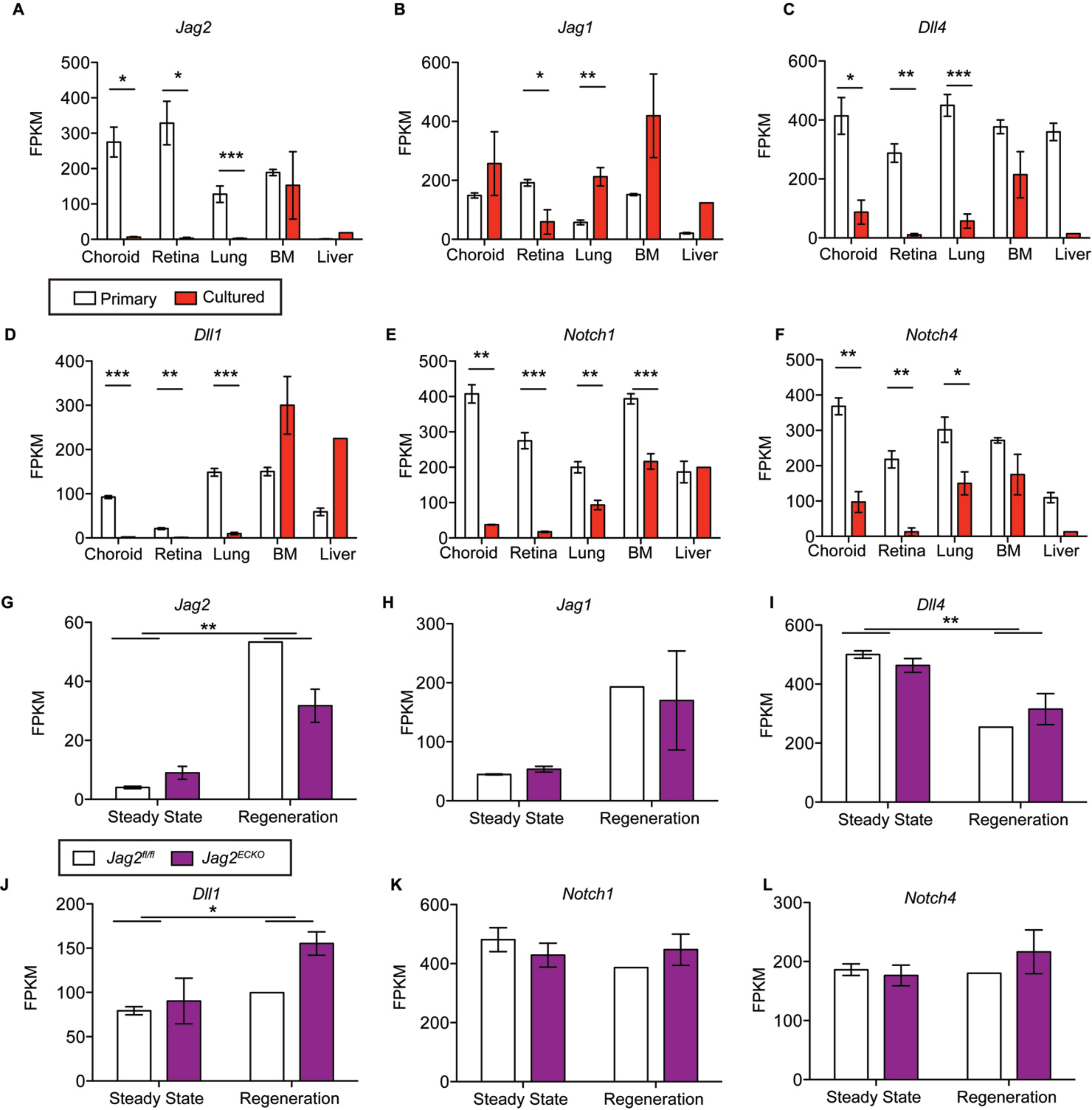

SUPPLEMENTARY FIGURE 2

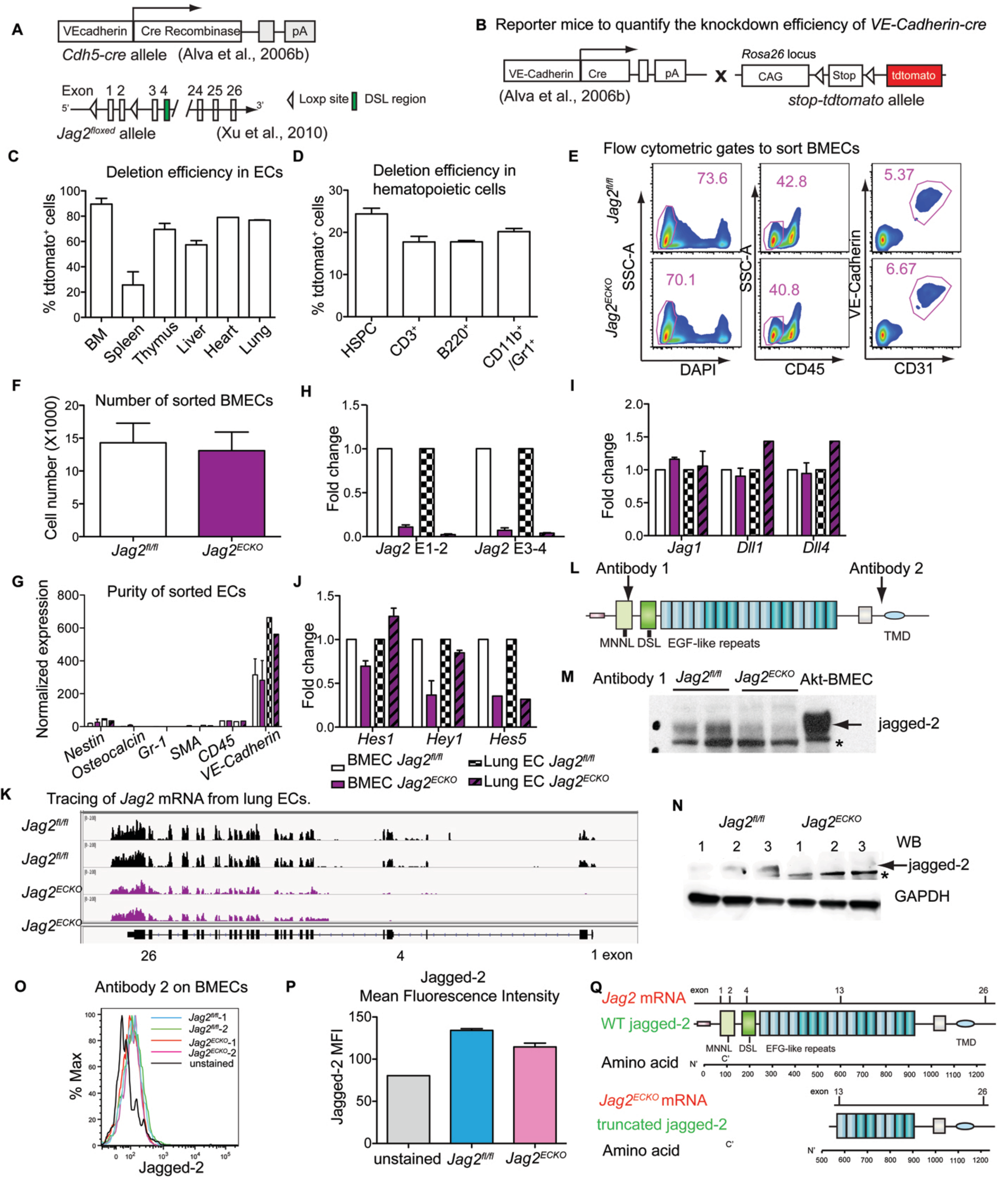

A. Full gel image for **Supplementary Figure 2M**

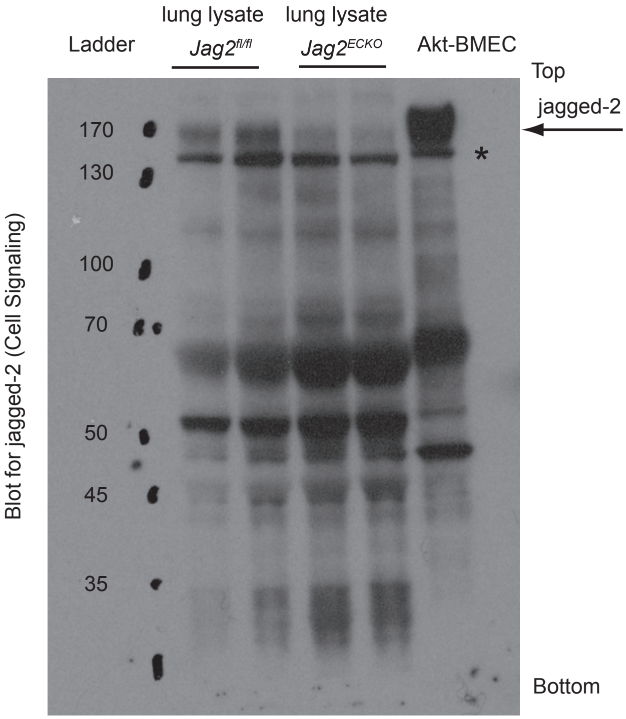

Loading amount: For lung lysate: 100 micrograms of total protein.
For Akt-BMEC: 25 micrograms of total protein.

* non-specific band

B. Full gel image for **Supplementary Figure 2N (jagged-2)**

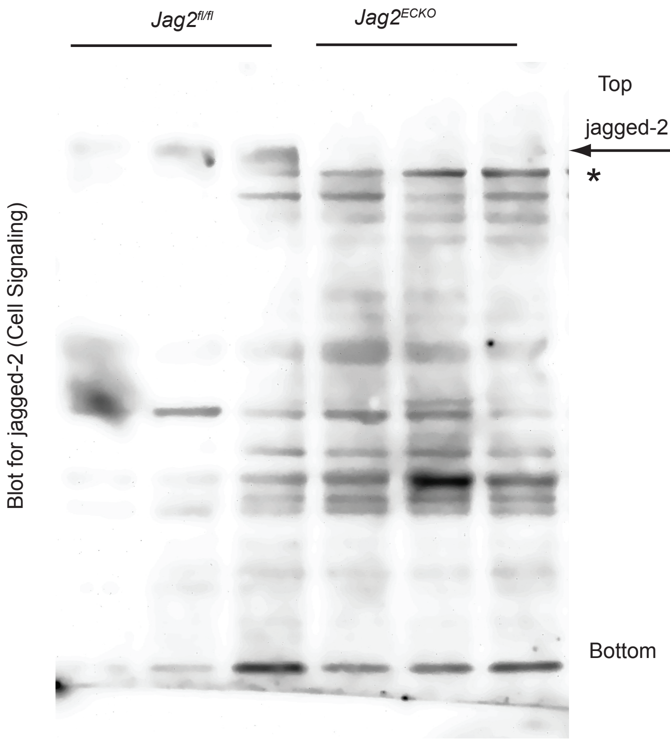

* non-specific band

C. Full gel image for **Supplementary Figure 2N (GAPDH)**

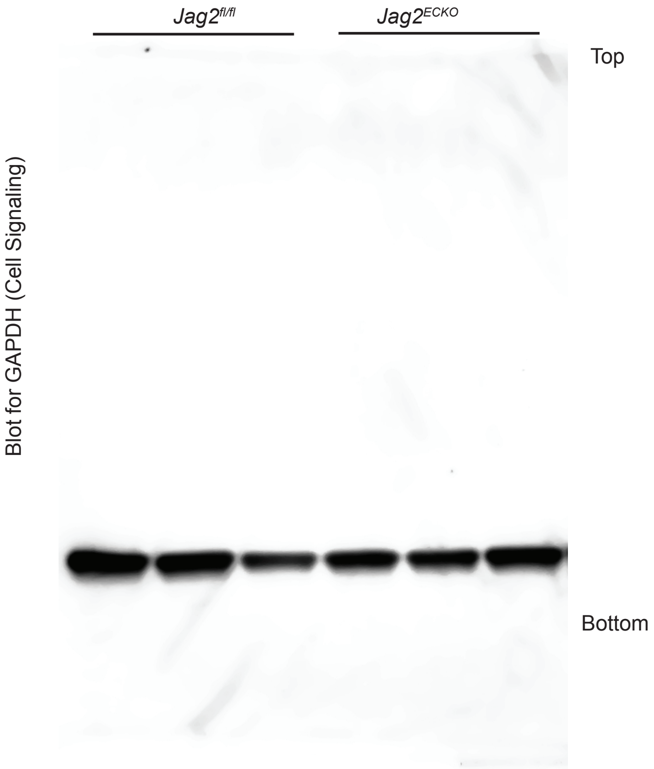

D. Half length gel image for jagged-2 expression in BMECs
Not shown in article. Related to reviewer B comment 8.

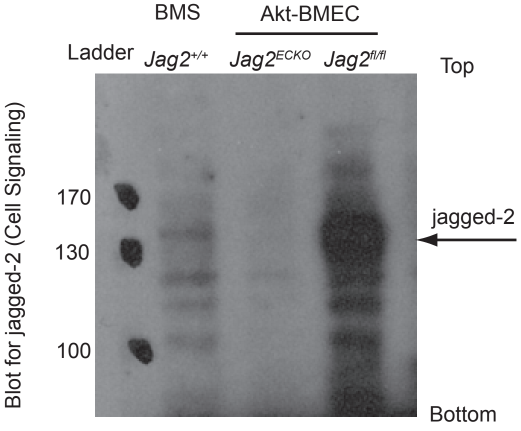

SUPPLEMENTARY FIGURE 3

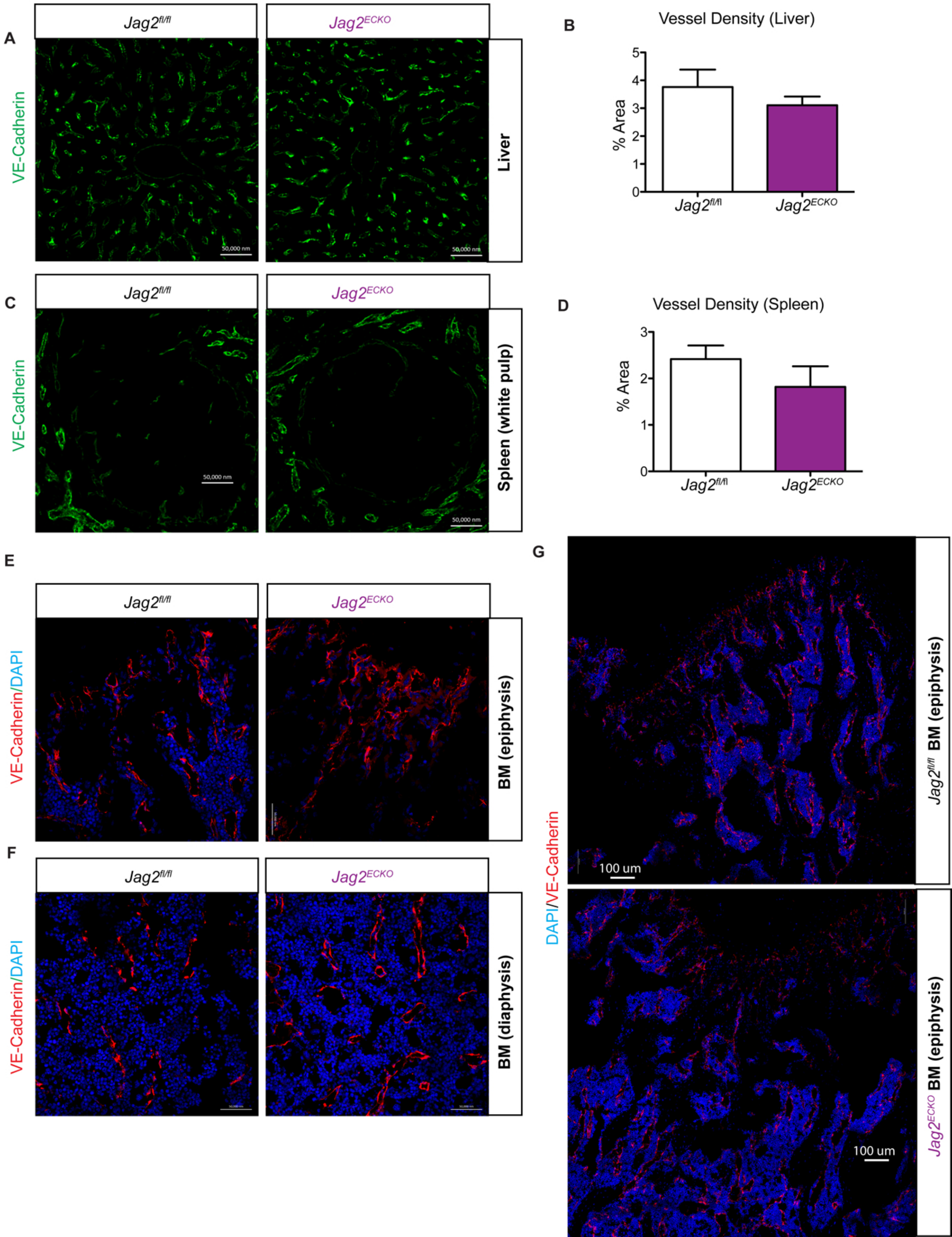

SUPPLEMENTARY FIGURE 4

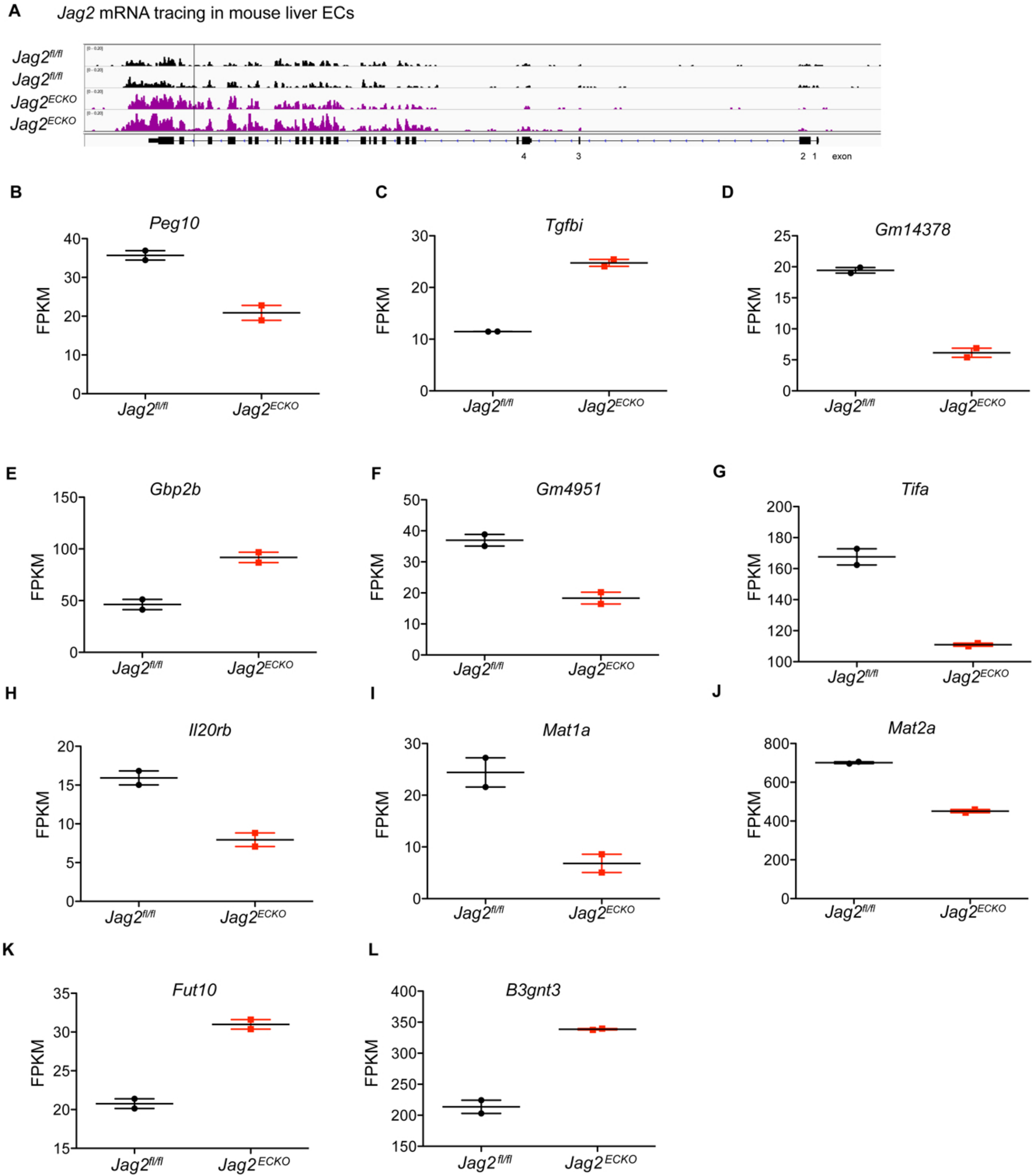

SUPPLEMENTARY FIGURE 5

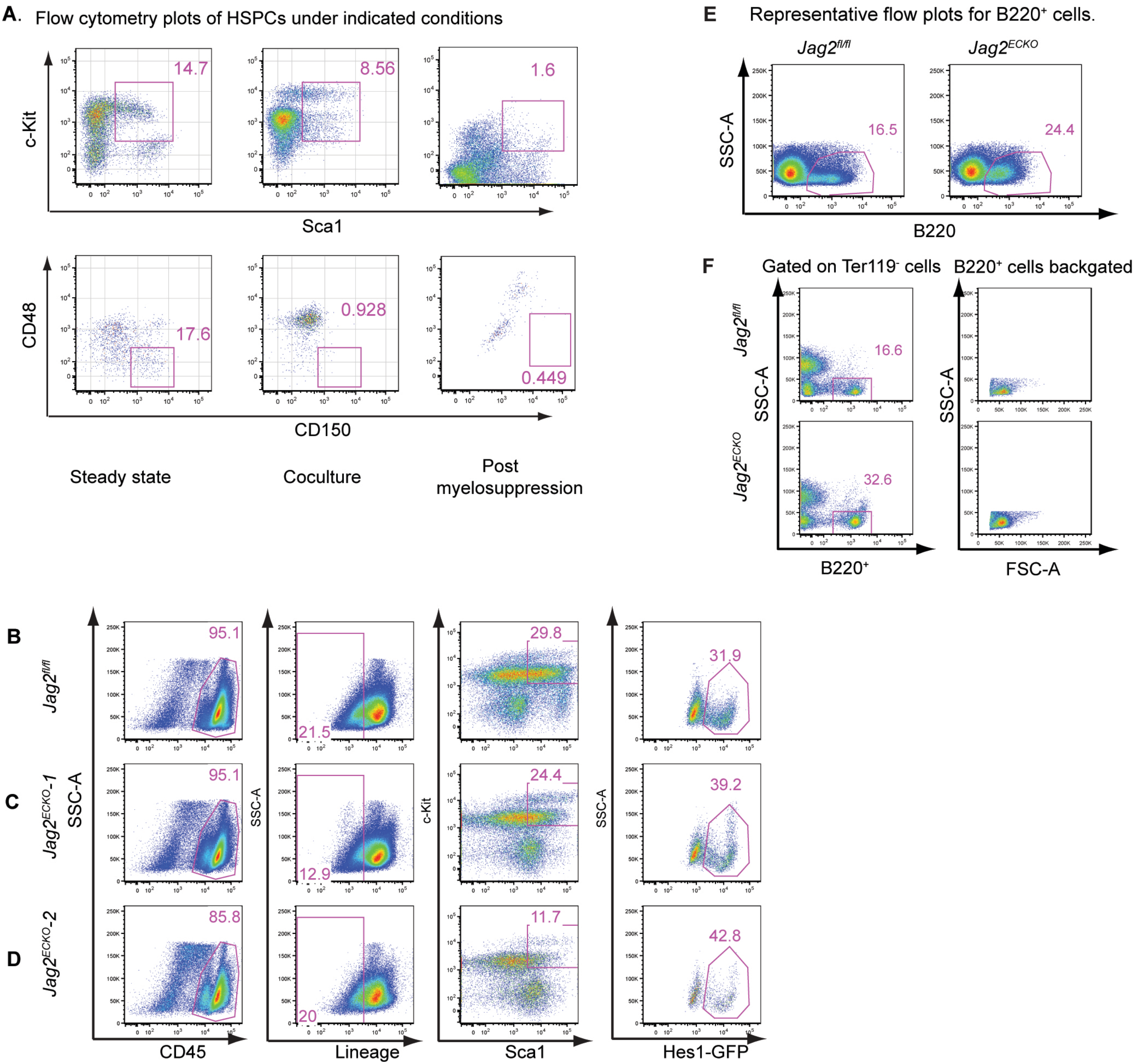

SUPPLEMENTARY FIGURE 6

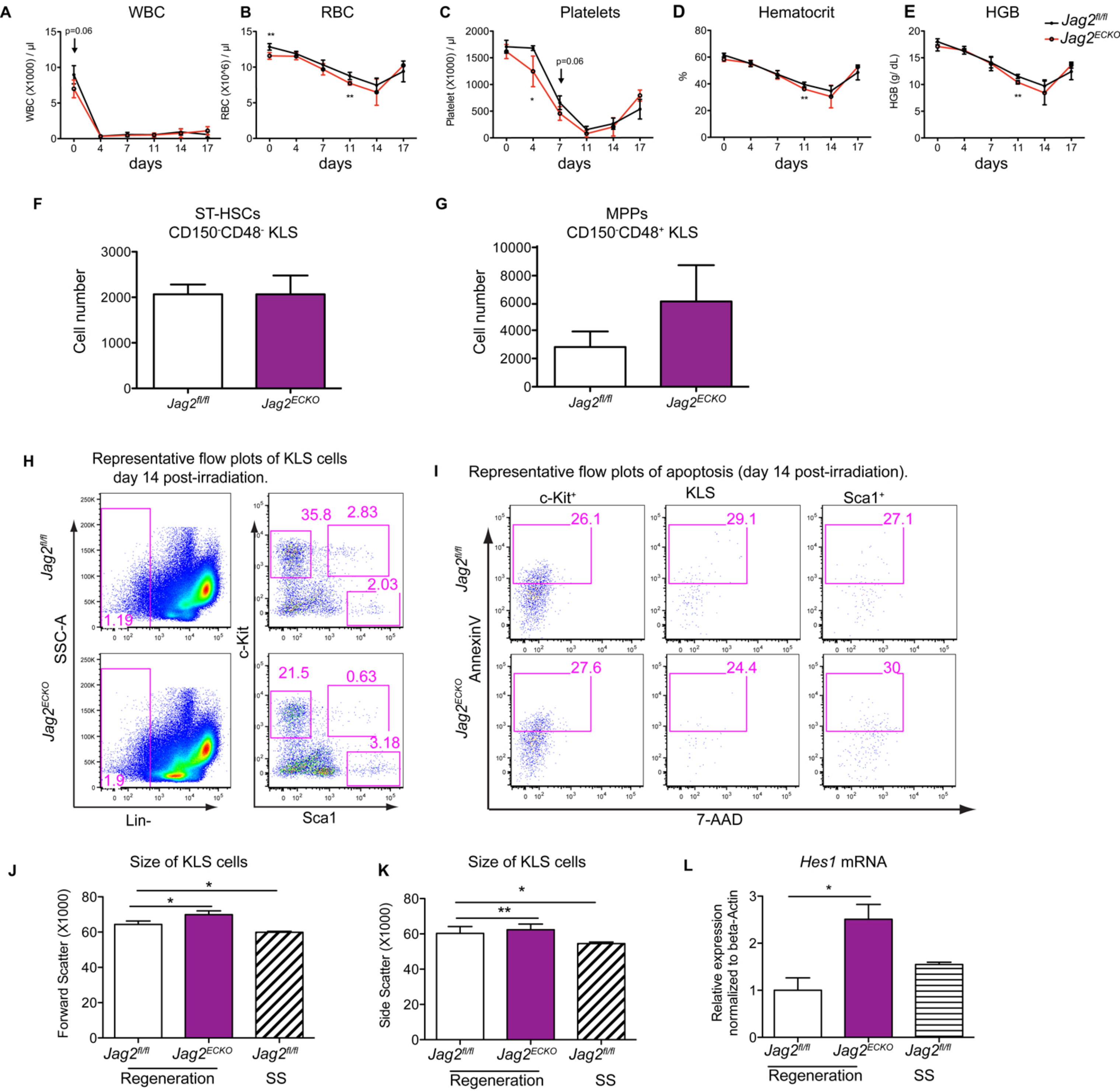

Supplementary Figure Legends

Supplementary Figure 1. Differential expression of Notch ligands and receptors in tissue-specific ECs.

A-F. The FPKM values of Notch ligands *Jag2*, *Jag1*, *Dll4*, *Dll1* and Notch receptors *Notch1* and *Notch4* in primary ECs compared with cultured ECs from indicated organs. Note that cultured ECs have undergone passages and were transduced with lentivirus encoding myristoylated *Akt1*. 2-6 biological replicates were used to generate the data, except for cultured liver ECs (n=1). **G-I**, At steady state or post myeloablative injury, the FPKM values of Notch ligands *Jag2*, *Jag1*, *Dll4*, *Dll1* and the Notch receptors *Notch1* and *Notch4* were shown for liver ECs from $Jag2^{fl/fl}$ or $Jag2^{ECKO}$ mice (relate to **Supplementary Figure 2** for $Jag2^{ECKO}$ mouse). For steady state, n=2 each of $Jag2^{fl/fl}$ and $Jag2^{ECKO}$ were used. For regeneration state, n=1 for $Jag2^{fl/fl}$ and n=3 for $Jag2^{ECKO}$ were used. The height of columns indicates the mean and error bars indicate S.E.M.. For **G-L**, the data points from the steady state were combined from the liver ECs of $Jag2^{fl/fl}$ and $Jag2^{ECKO}$ mice (n=4 in total), and the data points from the regeneration were combined from $Jag2^{fl/fl}$ and $Jag2^{ECKO}$ mice (n=4 in total), the p-value was then calculated between steady state and regeneration state. p-value was calculated using unpaired 2-tailed t-test. * $p<0.05$; ** $p<0.01$; *** $p<0.001$.

Supplementary Figure 2. Truncated *Jag2* mRNA and truncated jagged-2 protein are generated from ECs in $Jag2^{ECKO}$ mice, compared with $Jag2^{fl/fl}$ mice.

A. Schematic representation of *VE-Cadherin promoter-driven cre* recombinase transgenic line (17) and $Jag2^{floxed}$ line (18) used to generate EC-specific *Jag2* conditional deletion mice. **B**. The breeding strategies to quantify the deletion efficiency within ECs. Deletion efficiency of *VE-Cadherin-cre* (20) in ECs (**C**) from various organs, and hematopoietic cells in the BM (**D**) (n=3

biological replicates). **E**. Flow cytometric plots to sort out BMECs. **F**. The number of BMECs sorted from *Jag2*$^{fl/fl}$ and *Jag2*^{ECKO} mice (n=5 for each genotype). **G**. The purity of sorted ECs were examined using the following primers, *Nestin* for mesenchymal stromal cells, *Osteoclacin* for osteoblasts, *Gr-1* for granulocytes, *SMA* for smooth muscle cells, *CD45* for hematopoietic cells and *VE-Cadherin* for ECs (n=2). **H**. Deletion efficiency of *Jag2* mRNA at different regions. **I-J**, The mRNA expression level of Notch ligands *Jag1, Dll1*, *Dll4* and Notch targets *Hes1*, *Hey1* and *Hes5* in BMEC or lung microvascular ECs of *Jag2*$^{fl/fl}$ and *Jag2*^{ECKO} mice was quantified (n=3 for *Jag1*, n=2 for *Dll1*, *Dll4*, *Hes1* and *Hey1*). **K**. Tracing of *Jag2* mRNA in primary lung ECs from *Jag2*$^{fl/fl}$ and *Jag2*^{ECKO} mice (n=2 for each genotype). **L**. Schematic view of domain organization of full-length mouse jagged-2 protein and the two antibodies used to detect the N- or C-terminus region of jagged-2 protein. **M**. Using antibody 1, the whole tissue lysate of lungs was subjected to western blot analysis (n=2 for each genotype). **N**. Using antibody 1, the expression of N-terminus jagged-2 protein in Akt-BMECs from *Jag2*$^{fl/fl}$ and *Jag2*^{ECKO} mice was determined by western blot. **O**. Using antibody 2, the expression level of C-terminus jagged-2 was detected using flow cytometry (n=2 for each genotype). **P**. Quantification of mean fluorescence intensity (MFI) of jagged-2 expression in the BMECs. **Q**. Based on these data, a model of *Jag2* mRNA and protein expression was summarized. Error bars indicate S.E.M.. * in panel **M** and **N** indicated the non-specific bands. Please see the full, uncut western blot gels in the supplementary materials. The numbers in the flow plots represent percentages of cells.

Supplementary Figure 3. Loss of *Jag2* in ECs did not significantly alter the perfusion function and vascular patterning. The vasculature patterning of liver (**A**), spleen (**C**) and BM epiphysis (**E**), BM diaphysis regions (**F**) of *Jag2*$^{fl/fl}$ and *Jag2*^{ECKO} mice are shown. The

quantification of liver (**B**) and spleen (**D**) vasculature density (% VE-Cadherin$^+$ over area) are shown. For image panels in **A, C** and **E**, scales bar is 50 micrometers. **G**. Lower magnification of BM epiphysis region focusing on the tip of femur opposing the growth plate. Scale bars in G are 100 micrometers. Error bar is S.E.M.. For a, n=4 for *Jag2*$^{fl/fl}$, n=3 for *Jag2*^{ECKO}. For the rest of figure panels, n=3 for *Jag2*$^{fl/fl}$, n=2 for *Jag2*^{ECKO}. Mice were 6 weeks old, male.

Supplementary Figure 4. The altered molecular signatures of liver ECs upon loss of *Jag2*.
A. Tracing of *Jag2* mRNA in the RNA sequencing profile of liver ECs from *Jag2*$^{fl/fl}$ and *Jag2*^{ECKO} mice. **B-D**. Differentially regulated mRNAs that are related to the TGF-beta signaling pathway. **E-J**. Differentially expressed mRNAs that are related to the interferon gamma or TNF-alpha signaling pathways. **K,L**. Differentially expressed gene related to the cell-surface glycan modifications.

Supplementary Figure 5. BMEC-derived jagged-2 inhibits the differentiation of HSPCs into B cell lineage.
A. Flow cytometric plots of the HSPCs when freshly isolated from wild type mice (n=5), or during the coculture with BMECs (coculture) (n=3) and at day 16-post myeloablative injury of 650 cGy irradiation (post myelosuppression) (n=5). The flow cytometric plots for the post-myelosupression conditions (upper and lower panels) are from the same experiment as presented in **Fig. 6A** (first and second plots of the upper row). The expression pattern of CD150 versus CD48 within KLS cells indicated that coculture of Lin$^-$ cells on BMECs mimics the regeneration of KLS cells following myelosuppression. **B-D**. Representative flow plots for the gating of LSK HSPCs when Lin$^-$ cells were cultured with *Jag2*$^{fl/fl}$ BMECs, and two batches of *Jag2*^{ECKO} BMECs. *Jag2*^{ECKO}*-1* and *Jag2*^{ECKO}*-2* BMECs indicate BMECs from two different *Jag2*^{ECKO} mice. 3

biological replicates of Lin- cells were used to do the coculture experiments. The percentage of Hes1-GFP$^+$ cells among the KLS cells was also shown. In panel **B** and **D**, the third row from the left is the same flow plots presented in **Figure 3H** from left to right. **E**. The representative flow cytometric plots of B220$^+$ cells among the cocultured hematopoietic cells are shown. **F**. Representative flow plots for the B220$^+$ cells within the peripheral blood of 18 month-old *Jag2*$^{fl/fl}$ or *Jag2*^{ECKO} mice (n=7 for *Jag2*$^{fl/fl}$, n=8 for *Jag2*^{ECKO}). The numbers in the flow plots represent percentages of cells.

Supplementary Figure 6. Peripheral blood counts and apoptosis rate of BM cells during hematopoietic regeneration post myelosuppressive stress. Post 650 cGy sublethal irradiation, complete blood count (CBC) was quantified. The number of white blood cell count (WBC) (**A**), RBC count (**B**), platelets (**C**), hematocrit (**D**) and hemoglobin (HG) content (**E**) in the peripheral blood were quantified (n=4 each for *Jag2*$^{fl/fl}$ or *Jag2*^{ECKO}). (**F-G**) at day 16 post-irradiation, the number of ST-HSCs and MPPs was quantified. **H, I**. at day 14-post 650 cGy irradiation, the flow cytometric gating of hematopoietic populations and their apoptosis status in *Jag2*$^{fl/fl}$ or *Jag2*^{ECKO} mice. **J,K**. forward scatter and side scatter of KLS cells from *Jag2*$^{fl/fl}$ and *Jag2*^{ECKO} on day 21-22 post 650 cGy irradiation. SS indicates steady state conditions. **L**. *Hes1* mRNA expression level in the KLS cells on day 21-post 650 cGy irradiation (n=3 for *Jag2*$^{fl/fl}$; n=5 for *Jag2*^{ECKO} mice for Regeneration experiments; n=2 for SS experiments). Error bars indicate S.E.M.. p-value was calculated using unpaired 2-tailed student t-test. * $p<0.05$; ** $p<0.01$. The numbers in the flow plots represent percentages of cells.

Table 1. List of Antibodies used in this study

Antibody	*Clone number*	*Company*	*Note for applications*
**VE-Cadherin**	BV13	Biolegend	Flow Cytometry
**VE-Cadherin**	AF1002	R and D	IF
**CD31**	390	Biolegend	Flow Cytometry
**CD45**	30-F11	Biolegend	Flow Cytometry
**Ter119**	Ter119	Biolegend	Flow Cytometry
**B220**	RA3-6B2	Biolegend	Flow Cytometry
**CD3**	17A2	Biolegend	Flow Cytometry
**Gr-1**	RB6-85C	Biolegend	Flow Cytometry
**CD11b**	M1/70	Biolegend	Flow Cytometry
**CD41**	MWReg30	Biolegend	Flow Cytometry
**c-Kit**	2B8	Biolegend	Flow Cytometry
**Sca1**	D7	Biolegend	Flow Cytometry
**CD150**	TC15-F12.2	Biolegend	Flow Cytometry
**CD48**	HM48-1	Biolegend	Flow Cytometry
**CD34**	Mec14.7	Biolegend	Flow Cytometry
**Ki67**	B56	BD Biosciences	Flow Cytometry
**CD105**	MJ7/18	Biolegend	Flow Cytometry
**CD127**	A7R34	Biolegend	Flow Cytometry
**CD16/32**	93	Biolegend	Flow Cytometry
**Jagged2**	HMJ2-1	Biolegend	Flow Cytometry
**Jagged2 (Ab2)**	H-143	Santa Cruz	Flow Cytometry and Western blot
**Jagged2 (Ab1)**	C23D2	Cell Signaling	IF and western blot
**Jagged1**	polyclonal	abcam #85763	Western blot
**Dll1**	#2588 (catlog)	Cell Signaling	Western blot
**Dll4**	#2589 (catlog)	Cell Signaling	Western blot
**CD31**	Mec 13.3	Biolegend	Dynabeads isolation of endothelial cells
**GAPDH**	#2118 (catlog)	Cell Signaling	Western blot
**beta-actin**	AC-74	Sigma	Western blot

Table 2. List of primers used in this study

Primer name	*Sequence (5'-3')*
Jag2 E1-2 forward	GGCTGCTGCTGCTACTGGT
Jag2 E1-2 reverse	GCACACGCGCACGTACGTGTC
Jag2 E3-4 forward	CAATGACACCACTCCAGATGAG
Jag2 E3-4 reverse	CGCACGCTGGCATGATCAAC
Jag1 forward	AACGACCGTAATCGCATCGTACTGC
Jag1 reverse	CAGCCAAAGCCATAGTAGTGGTCATCAC
Dll1 forward	CCCATCCGATTCCCCTTCG
Dll1 reverse	GGTTTTCTGTTGCGAGGTCATC
Dll4 forward	TTCCAGGCAACCTTCTCCGA
Dll4 reverse	ACTGCCGCTATTCTTGTCCC
Notch1 forward	CCCTTGCTCTGCCTAACGC
Notch1 reverse	GGAGTCCTGGCATCGTTGG
Notch2 forward	GACTGCCAATACTCCACCTCT
Notch2 reverse	CCATTTTCGCAGGGATGAGAT
Notch3 forward	TGCCAGAGTTCAGTGGTGG
Notch3 reverse	CACAGGCAAATCGGCCATC
Notch4 forward	CTCTTGCCACTCAATTTCCCT
Notch4 reverse	TTGCAGAGTTGGGTATCCCTG
Hey1 forward	CCGACGAGACCGAATCAATAAC
Hey1 reverse	TCAGGTGATCCACAGTCATCTG
Hey2 forward	AAGCGCCCTTGTGAGGAAAC
Hey2 reverse	GGTAGTTGTCGGTGAATTGGA
Hes1 forward	CCAGCCAGTGTCAACACGA
Hes1 reverse	AATGCCGGGAGCTATCTTTCT
Sma forward	GTCCCAGACATCAGGGAGTAA
Sma reverse	TCGGATACTTCAGCGTCAGGA
Gapdh forward	AAATGGTGAAGGTCGGTGTGAACG
Gapdh reverse	GGTCAATGAAGGGGTCGTTGATGG
cre forward	ATGTCCAATTTACTGACCGTACACCA
cre reverse	ACGATGAAGCATGTTTAGCTGGCCCA
cre forward (FG)	GCGGTCTGGCAGTAAAAACTATC
cre reverse (FG)	GTGAAACAGCATTGCTGTCACTT
nestin forward	CCCTGAAGTCGAGGAGCTG
nestin reverse	CTGCTGCACCTCTAAGCGA-3
osteocalcin forward	TCTCTCTGACCTCACAGATGCCAAGC
osteocalcin reverse	AGCCATACTGGTCTGATAGCTCGTCAC
Gr-1 forward	GACTTCCTGCAACACAACTACC
Gr-1 reverse	ACAGCATTACCAGTGATCTCAGT
CD45 forward	TACACCCAGTGATGGTGTG
CD45 reverse	GCTGCTGAATGTCTGAGTG
VE-Cadherin forward	TGTGTTTTCGCACCAGGTATTCA
VE-Cadherin reverse	CAATGCTGAAATACTCATTTCCT

Hes5 forward	AGTCCCAAGGAGAAAAACCGA
Hes5 reverse	GCTGTGTTTCAGGTAGCTGAC
